# Evaluating the effect of upper-body morbidity on quality of life following primary breast cancer treatment: a systematic review and meta-analysis

**DOI:** 10.1007/s11764-023-01395-0

**Published:** 2023-05-18

**Authors:** Eliza R. Macdonald, Nadia M. L. Amorim, Amanda D. Hagstrom, Katarina Markovic, David Simar, Rachel E. Ward, Briana K. Clifford

**Affiliations:** 1 School of Health Sciences, Department of Exercise Physiology, UNSW, Sydney, Sydney, Australia; 2 Centre for Inflammation, Faculty of Science, School of Life Sciences, Centenary Institute and University of Technology Sydney, Sydney, NSW Australia; 3 School of Nursing, Midwifery and Social Work, The University of Queensland (UQ), Brisbane, Australia

**Keywords:** Breast cancer, Quality of life, Upper-body morbidity, Lymphoedema, Pain, Range of motion

## Abstract

**Purpose:**

Improvements in breast cancer management continue to increase survival and life expectancy after treatment. Yet the adverse effects of treatment may persist long term, threatening physical, psychological, and social wellbeing, leading to impaired quality of life (QOL). Upper-body morbidity (UBM) such as pain, lymphoedema, restricted shoulder range of motion (ROM), and impaired function are widely reported after breast cancer treatment, but evidence demonstrating its impact on QOL is inconsistent. Therefore, the aim of the study was to conduct a systematic review and meta-analysis evaluating the effect of UBM on QOL following primary breast cancer treatment.

**Methods:**

The study was prospectively registered on PROSPERO (CRD42020203445). CINAHL, Embase, Emcare, PsycInfo, PubMed/Medline, and SPORTDiscus databases were searched for studies reporting QOL in individuals with and without UBM following primary breast cancer treatment. Primary analysis determined the standardised mean difference (SMD) in physical, psychological, and social wellbeing scores between UBM + /UBM − groups. Secondary analyses identified differences in QOL scores between groups, according to questionnaire.

**Results:**

Fifty-eight studies were included, with 39 conducive to meta-analysis. Types of UBM included pain, lymphoedema, restricted shoulder ROM, impaired upper-body function, and upper-body symptoms. UBM + groups reported poorer physical (SMD =  − 0.99; 95%CI =  − 1.26, − 0.71; *p* < 0.00001), psychological (SMD =  − 0.43; 95%CI =  − 0.60, − 0.27; *p* < 0.00001), and social wellbeing (SMD =  − 0.62; 95%CI =  − 0.83, − 0.40; *p* < 0.00001) than UBM − groups. Secondary analyses according to questionnaire showed that UBM + groups rated their QOL poorer or at equal to, UBM − groups across all domains.

**Conclusions:**

Findings demonstrate the significant, negative impact of UBM on QOL, pervading physical, psychological, and social domains.

**Implications for Cancer Survivors:**

Efforts to assess and minimise the multidimensional impact of UBM are warranted to mitigate impaired QOL after breast cancer.

**Supplementary Information:**

The online version contains supplementary material available at 10.1007/s11764-023-01395-0.

## Introduction

With the advent of new and effective methods for detecting, diagnosing, and treating breast cancer, life expectancy following the completion of primary treatment is improving [1]. However, adverse cancer and treatment-related effects continue to arise over the course of treatment. If these persist, they stand to threaten physical, psychological, social, and spiritual wellbeing in the long term.

In the case of breast cancer, upper-body treatment modalities that target areas of the breast, chest, and axilla, leaving nearby musculoskeletal, lymphatic and neural structures vulnerable to injury or impairment [2, 3]. Surgery and radiation therapy to the breast and axillary or subclavicular lymph nodes can cause tissue scarring/fibrosis, axillary cording, and muscle tightness, leading to impaired shoulder kinetics, reductions in shoulder range of motion (ROM) [4], and pain or discomfort [5]. Damage to the lymphatic system can result in the development of breast or upper-limb lymphoedema, the accumulation of lymphatic fluid leading to extremity swelling [6, 7]. Nerve damage accrued during local treatment can lead to neuropathic pain, paraesthesia, and altered muscle activation [8, 9]. Systemic treatment is also implicated in the development of upper-body symptoms. Neurotoxic chemotherapy can induce peripheral neuropathy and manifest as pain or altered sensation in the distal extremities. Hormone therapies are known to cause arthralgia and myalgia, which may be experienced in the joints and muscles of the upper limb [10].

Treatment-related upper-body concerns may be acute, resolving with time after treatment [11, 12]. However, up to 51% of individuals report experiencing at least one upper-body symptom or limitation within 18 months following breast cancer treatment [13] and survivors of up to 10-years post-treatment report the presence of breast cancer-related lymphoedema [14], chronic somatic or neuropathic pain, restricted shoulder ROM, chemotherapy-induced peripheral neuropathy, or a combination of these [14–18].

Due to the prevalence and persistence of treatment-related upper-body morbidity (UBM), it is imperative to understand the impact of UBM on daily functioning and quality of life (QOL) long term, so that it can be suitably addressed [19–24]. However, substantial variation exists in the way that UBM is categorized — such as by type, cause, or severity [14] — the time at which UBM and QOL are assessed post-treatment [25], and the domains of QOL that are measured. As a result, the direction and magnitude of the effect of all types of UBM on multiple aspects of one’s life remains unclear. Given the volume and heterogeneity of studies reporting QOL and UBM after breast cancer, a meta-synthesis to elucidate the impact of UBM that persists beyond primary treatment on each domain of QOL is warranted. A greater understanding of the relationship between persisting UBM and QOL will help contribute to improving care provided after breast cancer treatment.

## Aim

The aim of this study was to conduct a systematic review and meta-analysis, to evaluate the effect of persistent UBM following primary breast cancer treatment, on multiple domains of QOL.

## Methods

The review was conducted in accordance with the PRISMA 2020 statement [26], and the Cochrane handbook for systematic review and meta-analysis [27]. The study was prospectively registered on PROSPERO (CRD42020203445).

CINAHL, Embase, Emcare, PsycInfo, PubMed/Medline, and SPORTDiscus databases were searched without language restrictions, from inception until 25 September 2020. Subject headings and keywords referencing breast cancer, QOL, and treatment-related UBM were employed in the search. A detailed search strategy is included in the supplementary materials (Online resource 1). The database search was repeated on 8 December 2021 and 7 March 2023.

Studies which met the following criteria were eligible for inclusion: (1) published in English language; (2) observational (cross-sectional or longitudinal) or interventional (outcomes of interest assessed prior to delivery of an intervention); (3) sample comprised of individuals who had completed primary treatment for breast cancer of any stage, type, and grade; (4) QOL reported in breast cancer survivors with and without UBM discretely, using validated, multidimensional QOL assessment tools.

Treatment-related UBM was defined as the presence of at least one of any upper-body symptom or limitation arising after breast cancer treatment, indicated by self-report or objective clinical assessment. The “condition” was dichotomised into UBM present (UBM +) or UBM absent (UBM −). Where studies grouped participants into UBM groups more than once—for example, on the basis of an interlimb circumference measure, and on the basis of self-report — QOL data were extracted based on the objective data categorisations of UBM + / − . If multiple UBM + or UBM − groups were present in one study – for example, lymphoedema *and* reduced shoulder ROM groups – QOL data were combined to create UBM + / − groups using Review Manager v5.4.1 (The Cochrane Collaboration) or provided by authors upon request.

Records were screened for eligibility in two stages and in duplicate. Title and abstract screening [EM (100%); KM (75%); BC (25%)] and full text screening [EM (100%); BC (50%); AH (50%)] were completed using the Rayyan systematic review web application (Rayyan Systems Inc) [28] and COVIDENCE systematic review software (Veritas Health Innovation) [29], respectively. Data from included articles were extracted in duplicate into predetermined spreadsheets by authors EM, BC, and NA. Where studies met inclusion criteria but UBM or QOL data could not be adequately extracted, authors were contacted and followed up via email.

Study quality was assessed in duplicate by EM, BC and NA using the Joanna Briggs Institute (JBI) Critical Appraisal Checklist for Analytical Cross-sectional Studies [30]. The checklist consists of eight criteria for assessing the risk of publication bias in included studies. As per the JBI Manual for Evidence Synthesis [31], reviewers determined a priori that studies which met ≥ 75% of the criteria would be considered “good” quality.

## Statistical analysis

Studies which presented QOL data (mean with variance), for UBM + and UBM − groups discretely, were included in the meta-analysis. Where QOL was assessed on multiple occasions, the measure taken at the latest timepoint post-treatment was included to capture the effect of persistent rather than acute UBM on QOL. Where the results of one study were reported across multiple publications, the record with the most complete dataset was included. Meta-analyses were conducted in Review Manager v5.4.1 (The Cochrane Collaboration) [32].

### Primary analysis

The primary meta-analyses evaluated the effect of UBM on (1) physical wellbeing, (2) psychological/emotional wellbeing, and (3) social wellbeing. Each analysis used a random effects model to determine the standardised mean difference (SMD) (95% confidence interval, significance *p* < 0.05) in continuous QOL scores from the relevant physical, psychological, or social domain. Within the three categories of the primary analysis, studies were further divided into subgroups according to QOL questionnaire. This was done to elucidate differences in the size and direction of the effect of UBM on QOL assessed using the different tools. Pooled effect sizes were categorised as small (SMD = 0.2), medium (SMD = 0.5), or large (SMD = 0.8) [33]. Studies reporting physical, psychological, and social wellbeing using multiple assessment tools were included once in each analysis for SMD, with preference for including scores from cancer-specific questionnaires.

In the sensitivity analyses, only studies with subjective reporting of UBM were included. This was done to elucidate if the effect of subjectively reported UBM on QOL differed significantly to that observed in the primary analysis (i.e. subjective and/or objective UBM). Sensitivity analysis including studies with objective reporting of UBM could not be completed due to data availability. Funnel plots for each of the primary analyses were generated in Review Manager (v5.4.1) (The Cochrane Collaboration) [32] to assess publication bias. Low publication bias was inferred when studies were evenly distributed either side of the main effect [27, 34].

### Exploratory analyses

Exploratory meta-analyses were performed with studies grouped according to the QOL assessment tool employed. These analyses used a random effects model to determine mean difference (MD) (95% confidence interval, significance *p* < 0.05) between UBM + and UBM − groups in QOL scores within the domains of each questionnaire. The mean difference between groups was compared to the questionnaire’s Minimal Clinically Important Difference (MCID) or Minimal Important Difference (MID), subject to their availability in the literature. The MCID and MID represent the minimum change in QOL score necessary for an individual to perceive an improvement or deterioration in wellbeing. Comparison to MID or MCID was completed to add clinical relevance to the results of the analysis, to improve the translation of findings into practice [27, 35].

## Results

**Fig. 1:**
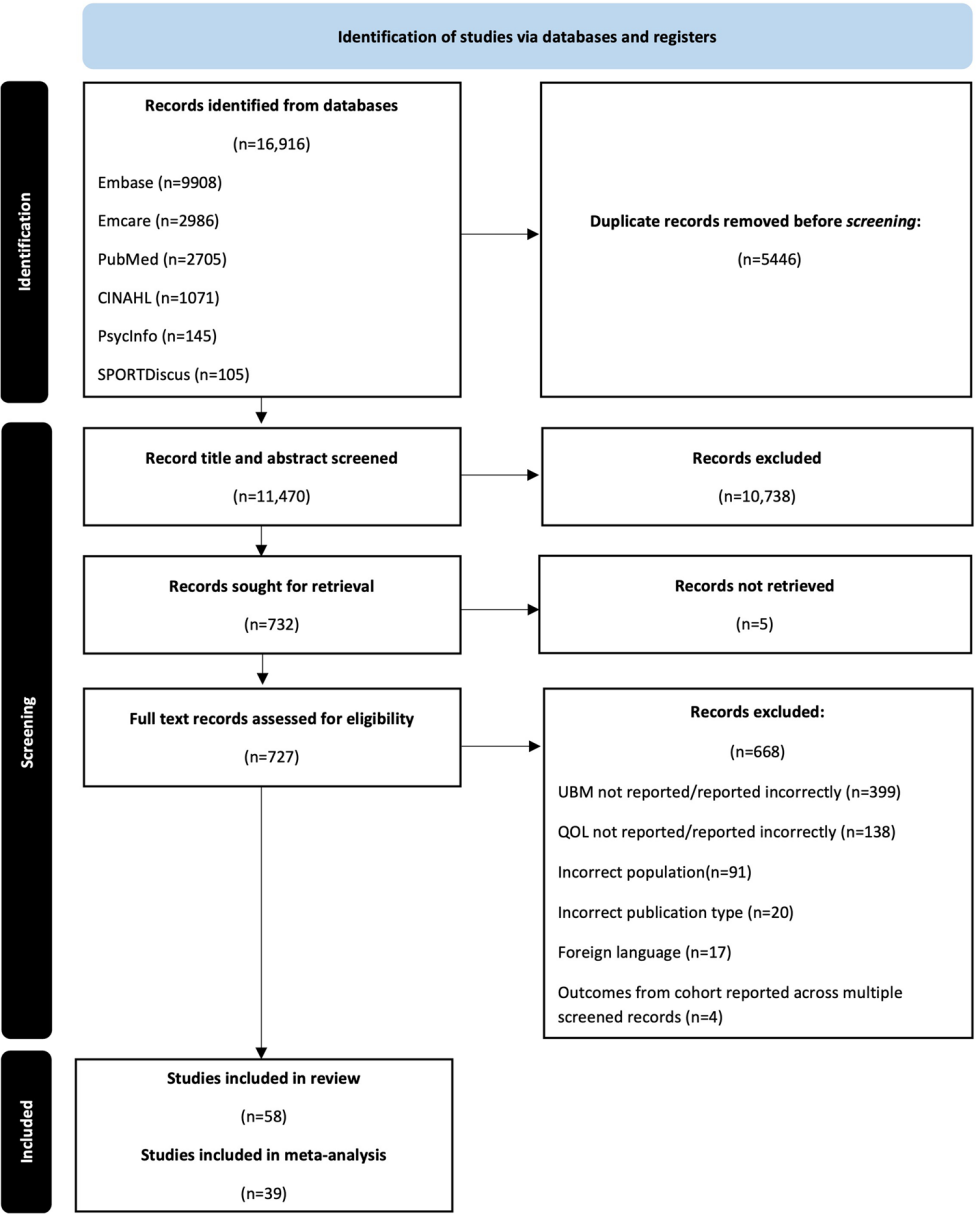
Prisma flow diagram for systematic review process [26]

The database search yielded 16,916 records. After duplicates were removed, 11,470 records were entered for title and abstract screening. Seven hundred and twenty-seven records were included for full-text screening from which a further 668 were excluded due to reasons outlined in Fig. 1. Fifty-eight records were included in the systematic review, of which 39 were suitable for inclusion in a meta-analysis. Four studies were reported across multiple publications [15, 24, 36, 37]. Results from the publication with the most complete dataset were included in analysis.

A summary of studies included in the systematic review can be found in Table 1. Types of UBM reported were lymphoedema (*n* = 31) of the upper-limb (*n* = 30) or breast (*n* = 1); chronic upper-body pain (*n* = 14), including post-mastectomy pain syndrome (*n* = 5), breast specific pain (*n* = 1), and lymphatic pain (*n* = 1); upper-body disability (*n* = 1); impaired shoulder ROM (*n* = 1); or a combination of upper-body symptoms and functional limitations (*n* = 11) (Table 1).

**Table 1:** Summary of findings

Author, date	Study type; Setting	Breast cancer diagnosis	Sample size (*n*)		Age (years)	Time of QOL assessment (years)
Aerts, 2011* [132]	Cross sectional; Outpatient clinic, The Netherlands	Stages 0–III	**Total**	**89**	**35–86** [*range]*	** > 2 years** **post-Sx**
			*UBM* +	59		
			*UBM* −	30		
Ahmed, 2008* [59]	Cross sectional; Mail survey, USA	Unilateral: In situ, local, regional/ distant	*UBM* +	*579*	*61(0.2)* *[Mean(SE)]*	*8.1(0.2)* years *post-Dx* *[Mean(SE)]*
			*UBM* −	*708*	*61(0.1)* *[Mean(SE)]*	*7.8(0.5)* years *post-Dx* *[Mean(SE)]*
Batenburg, 2023*^ [97]	Prospective cohort; Outpatient clinic; The Netherlands	Invasive; In situ	**Total**	**1613**	**58(24–84)** [Med (Range)]	**38(21–55) mo post-RT** [Med(IQR)]
			*UBM* +	*265*	*53(26***–***81)* *[Med* *(Range)]*	*38(21***–***55) mo post-RT* *[Med(IQR)]*
			*UBM* −	*1348*	*58(24***–***84)* *[Med* *(Range)]*	*38(21***–***56) mo post-RT* *[Med(IQR)]*
Beaulac, 2002* [60]	Cross sectional; Outpatient clinic, USA	Stages 0**–**II	*UBM* +	*42*	*61.1(12.7)* *[Mean (SE)]*	**–**
			*UBM* −	*109*	*62.9 (12.7) [Mean(SE)]*	**–**
Bell, 2014 [80]	Cohort study; Cancer registry, Australia	Primary invasive	*UBM* +	*424*	*53.7 (29.0,81.8) [Med (5th, 95th percentiles)]*	**5.7 (5.0, 6.7) years** *[Mean (5th, 95th percentiles)]*
			*UBM* −	*106*	*56 (32.7, 80.7)* *[Med (5th, 95th percentiles)]*	
Beyaz, 2016* [81]	Cross sectional study; Outpatient clinic, Turkey	**–**	**Total**	**131**	**55.2 (11.8)** *[Mean(SD)]*	** > 0.25 years** **post-Sx**
			*UBM* +	*84*	*54.2 (11.7) [Mean(SD)]*	
			*UBM* −	*47*	*57.1(11.9)* *[Mean(SD)]*	
Bulley, 2013 [133]	Cross sectional study; Outpatient clinic, UK	**–**	**Total**	**389**	**60.97(9.95)** *[Mean (SD)]*	**4.25 (0–30) years post-Tx** *[Med(range)]*
			*UBM* +	*102*		
			*UBM* −	*287*		
Bundred, 2020 [61]	Prospective cohort study; Hospital, UK	Invasive, grade 0**–**III	*UBM* +	*194*	*57.5 (11.7)* *[Mean (SD)]*	**2 years post-Tx**
			*UBM* −	*807*	*55.4(12.5)* *[Mean (SD)]*	
Caffo, 2003* [134]	Retrospective cross-sectional study; Outpatient clinic, Italy	In situ/invasive	**Total**	**568**	**60 (33–86)** *[Med* *(range)]*	**1–4 years post-Sx**
			*UBM* +	*210*	*57 (Med)*	
			*UBM* −	*319*	*61 (Med)*	
Carpenter, 1998 [82]	Cross sectional study; Outpatient clinic, USA	Stages 0**–**IIB	**Total**	**134**	**56.5(11)** *[Med (SD)]*	**2.92 (1.82) years post-Tx** *[Mean(SD)]*
			*UBM* +	*36*		
			*UBM* −	*70*		
Casso, 2004* [90]	Cross sectional study; Community, USA	In situ/invasive; stages 0**–**IV	**Total**	**216**	**45–60** *(Range)*	**7.2(5–10) years post-Dx** *[Mean(SD)]*
			*UBM* +	*80*		
			*UBM* −	*132*		
Chachaj, 2010 [62]	Cross sectional study; Oncology centre, Poland	**–**	*UBM* +	*117*	*61.39(9.44) [Mean(SD)]*	*6.3(3.68) years post-Sx [Mean(SD)]*
			*UBM* −	*211*	*59.95(10.56) [Mean(SD)]*	*7.35(7.19) years post-Sx [Mean(SD)]*
Dawes, 2008* [135]	Cross sectional study; Hospital, Canada	Stages I**–**II	*UBM* +	*16*	*62.4(11)* *[Mean (SD]*	**–**
			*UBM* −	*34*	*57.2(10)* *[Mean (SD)]*	**–**
DiSipio, 2009* [89]	Cross sectional study; Cancer registry, Australia	Infiltrating; Grades I**–**III	**Total**	**323**	** ≥ 50 (213)**	**1 year post-Dx**
			*UBM* +	*160*		
			*UBM* −	*141*		
Engel, 2003 [91]	Prospective cohort study; Community, Germany	Stages 0**–**IV	*UBM* +	*97*	**–**	**5 years post-Tx**
			*UBM* −	*160*	**–**	
Fu, 2022 [136]	Cross-sectional study; Outpatient BC clinic; USA		**Total**	**345**	**59(26–82)** (Med [Range])	**3(0–43) years post-Dx**
			*UBM* +	*215*	**–**	**–**
			*UBM* −	*139*	**–**	**–**
Gong, 2020* [84]	Retrospective cohort study; Hospital, China	Early stage or advanced stage	*UBM* +	*560*	> *35 (472)*	** > 0.25 years** **post-Sx**
			*UBM* −	*1423*	> *35 (1321)*	
Hamood, 2018* [83]	Cross sectional cohort study; Community health fund, Israel	Early stage or regionally advanced	*UBM* +	*305*	*63.8(13.9) [Mean(sd)]*	*7.9(3.2) years post-Dx [Mean(sd)]*
			*UBM* −	*105*	*68.9(12.9) [Mean(sd)]*	*9.37(3.4) years post-Dx [Mean(sd)]*
Hau, 2013 [64]	Cohort study; Hospital, Australia	Stages 0**–**II	**Total**	**428**	**58(24–81)** *[Mean(range)]*	**10 years post-Sx**
Hayes, 2022^ [137]	Prospective Cohort study; Community, USA	Stages I**–**III	**Total**	**2442**	** < 50 (1189)** ** ≥ 50 (1253)**	**25(20–36) mo post-Dx** (Med [Range])
Heiney, 2007* [65]	Cross sectional study, USA	Stages 0**–**IV	*UBM* +	*122*	*58.7(11.2) [Mean(sd)]*	*4.03(0.63***–***13.96) years post-Dx* *[Med (range)]*
			*UBM* −	*415*	*61.0(11.1)* *[Mean (sd)]*	*2.93 (0.13***–***15.31) years post-Dx* *[Med (range)]*
Hickey, 2011 [138]	Retrospective cohort study; Hospital, Ireland	**–**	*UBM* +	*18*	*46.4(34.0***–***58.0) [Med (range)]*	*2.13 (0.25***–***7) years post-Tx* *[Med (range)]*
			*UBM* −	*24*	*52.5(38.0***–***75.0) [Med* *(range)]*	*3 (0.67***–***7) years post-Tx* *[Med (range)]*
Hormes, 2010* [66]	Randomised control trial; University, USA	**–**	**Total**	**295**	**55.98(8.83)** *[Mean (sd)]*	**1–15 years post-Dx**
			*UBM* +	*148*		
			*UBM* −	*145*		
Jariwala, 2022* [92]	Cross sectional study; University, India	Stages I**–**III	**Total**	**212**	**50(13.7)** *[Mean(sd)]*	**2.7 years post-Sx**
			*UBM* +	*104*		
			*UBM* −	*108*		
Jørgensen, 2021* [139]	Cross sectional study: Breast Cancer Registry, Denmark	In situ/invasive	**Total**	**1067**	**64–35(10.23)** *[Mean(sd)]*	**7.95(3.67) years post-Sx** *[Mean* *(sd)]*
			*UBM* +	*244*	*59.73(9.85)* *[Mean(sd)]*	
			*UBM* −	*823*	*65.51(9.99)* *[Mean(sd)]*	
Kaur, 2017* [85]	Cross-sectional cohort study; Hospital, India	Stages I**–**III	**Total + **	***210***	*57 (Med)* *61 (Med)*	** > 0.25 years** **post-Sx**
			*UBM* −	*319*		
			*UBM* −	*33*		
Kibar, 2017* [140]	Cross-sectional cohort study; Hospital, Turkey	Unilateral BC	**Total**	**201**	**52.5(10.4)** *[Mean(sd)]*	**0.66 (3.98) years post-Tx** *[Mean(sd)]*
			*UBM* +	*107*	*53.4(10.3) [Mean(sd)]*	
			*UBM* −	*94*	*51.5(10.5) [Mean (sd)]*	
Koca, 2020* [141]	Cross sectional cohort study; Oncology outpatient clinic, Turkey	Stages I**–**IV	**Total**	**67**	**30.4(11.2)** *[Mean (sd)]*	**3 (0.17–20) post-Sx** *[Med(range)]*
			*UBM* +	*15*	*52.37(11.21) [Mean (sd)]*	
			*UBM* −	*51*	*49.3(10.9)* *[Mean (sd)]*	
Koehler, 2020 [63]	Prospective cohort study; Community Dragon Boating Festival, USA	Stages 0**–**IV	**Total**	**757**	**–**	**9 (5, 14) years post-Sx** *[Med(95%CI)]*
			*UBM* +	*293*	**–**	*10(6,14) years post-Sx* *[Med(95%CI)]*
			*UBM* −	*464*	**–**	*9(5,14) years* *post-Sx* *[Med(95%CI)]*
Kwan, 2002 [142]	Cross-sectional cohort study: Community mailout, Canada	In situ or invasive BC	*UBM* +	*61*	**––**	*2***–***7 years post-Dx*
			*UBM* −	*51*	**–**	
Langford, 2015* [143]	Prospective cohort study; Hospital and community, USA	Stages 0**–**IV	*UBM* +	*158*	*54.8(11.9) [Mean(sd)]*	*0.08 years post-Sx*
			*UBM* −	*122*	*58.7(11.2) [Mean(sd)]*	
Lee, 2012* [144]	Prospective cohort study; Hospital, Korea	Stages I**–**IV	*UBM* +	*58*	*54.1(10.8) [Mean(sd)]*	*3.69 (2.06) years post-Sx [Mean(sd)]*
			*UBM* −	*38*	*51.82(9.84) [Mean(sd)]*	*3.31(2.16) years post-Sx [Mean(sd)]*
Lopez-Penha, 2014* [67]	Prospective cohort study; The Netherlands	Stages 0**–**IV	*UBM* +	*26*	*55.4 (11.1) [Mean(sd)]*	*6.42 (0.83) years post- Tx [Mean(sd)]*
			*UBM* −	*119*	*56.5(11.3) [Mean(sd)]*	*6.30 (0.80) years post- Tx [Mean(sd)]*
Macdonald, 2005* [86]	Cohort study; Hospital, UK	**–**	**Total**	**103**	**62(10.5)** *[Mean(sd)]*	**7–12** years** post-Sx**
			*UBM* +	*59*	*49.5(9.8) [Mean(sd)]*	*8.9(1.9) years post-Sx [Mean(sd)]*
			*UBM* −	*54*	*56.2(10.9) [Mean(sd)]*	*9.1(1.8) years post-Sx [Mean(sd)]*
Mak, 2009* [68]	Cross sectional case control study; Hospital, Australia	Stages I**–**III	*UBM* +	*101*	*53.0 (9.6) [Mean(sd)]*	*3.7(2.2) years post-Sx [Mean(sd)]*
			*UBM* −	*101*	*50.3(7.7) [Mean(sd)]*	*3.5(2) years* *post-Sx [Mean(sd)]*
Mandelblatt, 2002* [145]	Longitudinal cohort study; Hospital, USA	Stages I-IIB	**Total**	**571**	** ≥ 67**	**2 years post-Tx**
			*UBM* +	*219*		
			*UBM* −	*352*		
Meijuan, 2013* [87]	Cross sectional study; Hospital, China	**–**	**Total**	**225**	**53 (29–74)** *[Mean* *(Range)]*	**1–3.3 years** **post-Sx**
			*UBM* +	*62*		
			*UBM* −	*163*		
Mülkoğlu, 2021 [146]	Cross sectional study: Hospital LE clinic, Turkey	Invasive BC	*UBM* +	*25*	*48(6)* *[Mean(sd)]*	*5.5 (3.0) years post-Sx*
			*UBM* −	*20*	*48.8(4.8)* *[Mean(sd)]*	*4.0(2.0) years post-Sx*
Nesvold, 2011* [147]	Cross sectional study; Hospital, The Netherlands	Stage II	*UBM* +	*80*	*54.6(7.7) [Mean(sd)]*	*4.4(1.4) years post-Sx*
			*UBM* −	*175*	*54.5(8.2) [Mean(sd)]*	*3.9(0.8) years post-Sx*
Neuner, 2014 [79]	Population based longitudinal study; Community, USA	In situ, localised, regional/remote	**Total**	**3083**	**72.5(5** − **3)** *[Mean(sd)]*	**5 years post-Dx**
			*UBM* +	*518*		
			*UBM* −	*2565*		
Oliveri, 2008* [148]	Cross-sectional study: CALGB research institutions, USA	**–**	**Total**	**245**	**63(10)** *[Mean(sd)]*	**12.5(9.4** − **16.5) years post-Dx** *[Mean* *(range)]*
			*UBM* +	*75*	*61(9) [Mean(sd)]*	*12.4(9.4* − *16.4) years post-Dx [Mean* *(range)]*
			*UBM* −	*170*	*63(10) [Mean(sd)]*	*12.6(9.4* − *16.5) years post-Dx* *[Mean* *(range)]*
Pinto, 2013* [100]	Cross-sectional study: Outpatient clinic, Italy	Stages I − II	*UBM* +	*50*	*61.8(10.18) [Mean(sd)]*	*7.66(3.68) years post-Sx [Mean(sd)]*
			*UBM* −	*50*	*61.26(10.18) [Mean(sd)]*	*7.26(3.43) years post-Sx [Mean(sd)]*
Popovic-Petrovic, 2018* [150]	Cross-sectional study; Oncology institute, Serbia	–	*UBM* +	*34*	*60.2(8.82) years [Mean(sd)]*	−
			*UBM* −	*30*	*56.16(10.18) years [Mean(sd)]*	−
Pyszel, 2006 [69]	Cross sectional study: Community group survey, Poland	−	*UBM* +	*84*	*75(40* − *77) years [Med* *(range)]*	−
			*UBM* −	*181*	*57(31* − *80) years [Med* *(range)]*	−
Recchia, 2005* [151]	Cross sectional study: Hospital, Brazil	DCIS, Invasive: Early -advanced	*UBM* +	*15*	**51.23(8.72) years [Mean(sd)]**	**5 years post-Tx**
			*UBM* −	*15*		
Ridner, 2005* [70]	Cross sectional study: Community, USA	Stages 0 − III	**Total**	**128**	−	**6.08(3.83) years post-Dx** *[Mean(sd)]*
			*UBM* +	*64*	*58(10.2) years [Mean(sd)]*	*6.83(3.92) years post-Dx [Mean(sd)]*
			*UBM* −	*64*	*55 (8.9) years [Mean(sd)]*	*5.5(3.67) years post-Dx [Mean(sd)]*
Round, 2006 [71]	Cross sectional study: Cancer registry, Australia	Invasive BC Grades I − III	**Total**	**287**	** < 45 (51)** **45** − **54 (98)** **55** − **64 (86)** ** ≥ 65 (52)**	** < 0.5 years post-Dx**
			*UBM* +	*78*		
			*UBM* −	*205*		
Speck, 2010* [152]	Randomised control trial; Community, USA	Stages 0 − III	*UBM* +	*112*	*57.04(9.02) [Mean (sd)]* *(Tx* + *CG)*	*6.98(3.64) years post-Dx [Mean(sd)]* *(Tx* + *CG)*
			*UBM* −	*122*	*56.04(7.57) [Mean (sd)]* *(Tx* + *CG)*	*3.3(1.22) years post-Dx [Mean(sd)]* *(Tx* + *CG)*
Sürmeli, 2019* [153]	Cross-sectional study; Turkey	−	*UBM* +	*27*	*52.78(7.65) [Mean(SS)]*	−
			*UBM* −	*29*	*50.62(7.25) [Mean(SS)]*	−
Tan, 2023 [154]	Prospective cohort study; Outpatient hospital, USA		**Total**	**210**	**51.4(13.1)** *[Mean(SD)]*	−
			*UBM* +	*135*	*49.9(12.9)* *[Mean(SD)]*	−
			*UBM* −	*75*	*54.1(13.0)* *[Mean(SD)]*	−
Togawa, 2021* [72]	Prospective cohort study; Cancer registry, USA	Stages 0 − IIIA	**Total**	**499**	**38** − **49(128)** **50** − **59(215)** **60** − **69(146)**	**3.33 years** **post-Dx**
			*UBM* +	*137*	*38* − *49(45)* *50* − *59(59)* *60* − *69(33)*	
			*UBM* −	*362*	*38* − *49(83)* *50* − *59(156)* *60* − *69(123)*	
Vassard, 2010 [102]	Randomised control trial: Rehabilitation centre, Denmark	Stages I − III	*UBM* +	*125*	< *45 (26%);* *45* − *55 (44%); 55* − *65(22%);* > *65(9%)*	< *1* year* (51);* > *1* year* (49)* *post-Sx*
			*UBM* −	*508*	< *45 (22%);* *45* − *55 (39%); 55* − *65(29%);* > *65(10%)*	< *1* year* (65);* > *1* year* (35)* *post-Sx*
Velanovich, 1999 [73]	Cross sectional study: Hospital, USA	−	*UBM* +	*11*	*59.1(11.7) [Mean(sd)]*	−
			*UBM* −	*45*	*62.8(12.7) [Mean(sd)]*	−
Wilson, 2005* [74]	Cross sectional study: Hospital, USA	Early stage	*UBM* +	*32*	*50.6(10.2) [Mean(sd)]*	*2.6(2.1) years post-Dx [Mean(sd)]*
			*UBM* −	*78*	*52.8(9.1) [Mean(sd)]*	*2.1(1.7) years post-Dx [Mean(sd)]*
Young-Afat, 2019* [75]	Longitudinal cohort study; Hospital, The Netherlands	Stages 0 − ≥ III	**Total^**	**836**	**58(16)** *[Mean (IQR)]*	−
			*UBM* +	*33*	−	*3 years post-RT*
			*UBM* −	*568*	−	*3 years post-RT*
Yusof, 2021a* [76]	Cross sectional study; Community survey, Malaysia	Stages I − IV	**Total**	**113**	**51.04(8.63)** *[Mean(sd)]*	**5.5(4.6) years post-Dx** *[Mean(sd)]*
			*UBM* +	*30*	−	−
			*UBM* −	*83*	−	−
Yusof, 2021b* [77]	Case control study; Malaysia	Stages I − IV	**Total**	**160**	**51.04(8.63)** *[Mean(sd)]*	**5.64(4.34) years post-Dx**
			*UBM* +	*33*	*51.73(8.15)* *[Mean(sd)]*	*5.3(4.10) years post-Dx* *[Mean(sd)]*
			*UBM* −	*127*	*45.23(8.35)* *[Mean(sd)]*	*5.72(4.40) years post-Dx* *[Mean(sd)]*
*Zhao, 2020 *[78]	Cross-sectional study; Hospital, China	Stages 0 − IV	*UBM* +	*155*	*30* − *39 (8)* *40* − *49 (64)* *50* − *59 (64)* ≥ *60 (19)*	*1.58(0.83* − *2.92) years post-Dx [Med(IQR)]*
			*UBM* −	*90*	≤ *29 (3)* *30* − *39 (10)* *40* − *49 (46)* *50* − *59 (22)* ≥ *60 (9)*	*1.33(1.08* − *1.75) years post-Dx [Med(IQR)]*

Author, date	Treatment type				UBM type(s), criteria	QOL assessment tool & summary of findings
	Sx (*n*)	RT (*n*)	CT (*n*)	ET (*n*)		
Aerts, 2011* [132]	**89**	**60**	**–**	**36**	**Restricted shoulder ROM** > 10° difference between sides, any direction	**WHOQOL-BREF** QOL ↓ with UBM + for physical^++^ and psychological^++^ health QOL UBM + ↔ UBM − for general, health, social relationships, and environmental health
	*59*	**–**	**–**	**–**		
	*30*	**–**	**–**	**–**		
Ahmed, 2008* [59]	*575*	*Br(199)* *Ax(70)*	*76*	*36*	**LE or UB symptoms** Reported Dx of LE or symptoms on validated questionnaire	**SF-36** QOL ↓ with arm symptoms across all subscales^+++.^ QOL ↓ with LE for all domains^+^ excl. mental health (*p* = 1.00) and role functioning, emotional (*p* = 0.054) LE ↔ arm symptoms for all subscales
	*701*	*Br(233) Ax(56)*	*69*	*310*		
Batenburg, 2023*^ [97]	**1576**	**Br(1163)** **Ax(450)**	**666**	**–**	**UB Symptoms** Mod-severe breast or chest wall pain + 1 of arm/breast LE; breast firmness; ↓ arm movement on self-reported questionnaire	**EORTC QLQ-C30** QOL ↓with UBM across physical functioning, social functioning, and role functioning. No statistical analysis presented
	*265*	*Br(171)* *Ax(94)*	*144*	**–**		
	*1348*	*Br(992)* *Ax(356)*	*521*	**–**		
Beaulac, 2002* [60]	*42*	*22*	*13*	**–**	**LE** Arm water volume displacement > 200cm^2^ on affected side	**FACT-B** QOL ↓ with UBM + for physical, functional, and emotional wellbeing, BC subscale, and total FACT-B scores^+++^
	*109*	*58*	*33*	**–**		
Bell, 2014 [80]	*423*	*254*	*329*	*143*	**Breast pain** Self-reported/questionnaire	**PGWB** QOL ↓ with UBM + for anxiety^+^, general health^+^, and total QOL^+^ scores UBM + ↔ UBM − for depressed mood, positive wellbeing, self-control, and vitality subscales
	*105*	*84*	*49*	*30*		
Beyaz, 2016* [81]	**131**	**93**	**120**	**81**	**PMPS** Pain at breast, chest, scar tissue, arm, or axilla > 3 months post-Sx on VAS, DN-4, McGill pain questionnaire	**SF-36** QOL ↓ with UBM + across all SF-36 subscales^++^
	*84*	*66*	*77*	*51*		
	*47*	*27*	*43*	*30*		
Bulley, 2013 [133]	**383**	**Br(317)** **Ax(94)**	**–**	**–**	**LE** Perometry interlimb volume difference > 10%	**FACT-B + 4** QOL ↓ with UBM + for arm symptoms subscale only^+++^ UBM + ↔ UBM − for physical, family/social, emotional, functional wellbeing, BC subscale and trial outcome index
Bundred, 2020 [61]	*194*	*168*	*135*	*151*	**LE** Relative arm volume increase (RAVI) > 10%	**FACT-B + 4** QOL ↓ with UBM + for arm symptom subscale^+++^ and trial outcome index^++^ UBM + ↔ UBM − for FACT-B total score
	*807*	*644*	*523*	*663*		
Caffo, 2003* [134]	**568**	**481**	**221**	**–**	**Chronic upper-body pain** McGill Pain questionnaire	**20-item,** **multi-dimensional QOL tool** QOL ↓ with UBM + for physical wellbeing^+++^, physical autonomy^+++^, personal relationships^+++^, and psychological wellbeing^+++^
	*210*	**–**	**–**	**–**		
	*319*	**–**	**–**	**–**		
Carpenter, 1998 [82]	**134**	**55**	**60**	**62**	**PMPS** Brief pain inventory (BPI)	**SF-12** QOL ↓ with UBM + for physical^+++^ and mental^+^ component scores
	*36*	*22*	*17*	**–**		
	**–**	**–**	**–**	**–**		
Casso, 2004* [90]	**216**	**136**	**119**	**80**	**Breast symptoms: *Pain, swelling, numbness, other*** Self-report questionnaire	**SF-36** QOL ↓ with UBM + in all subscales^++^ and mental^++^ and physical^++^ component scores
Chachaj, 2010 [62]	*117*	*64*	*65*	*82*	**LE** Self-reported LE, confirmed by interlimb circumference difference ≥ 2 cm	**EORTC QLQ-C30** QOL ↓ with UBM + for global QOL ^++^
	*211*	*86*	*131*	*147*		
Dawes, 2008* [135]	*16*	**–**	**–**	**–**	**LE** Interlimb volume difference ≥ 200 ml	**SF-36; EORTC QLQ-C30; EORTC QLQ-BR23** UBM + ↔ UBM − for SF-36 scores UBM + ↔ UBM − for EORTC QLQ-C30/BR23 scores
	*34*	**–**	**–**	**–**		
DiSipio, 2009* [89]	**323**	**151**	**159**	**–**	**UB disability** DASH score ≥ 11	**FACT-B + 4** QOL ↓ with UBM + for Total FACT-B + 4 score^+++^
Engel, 2003 [91]	*97*	**–**	**–**	**–**	**Arm morbidity** Questionnaire response indicating the presence of any one of: Arm swelling; Limitations in arm movement	**EORTC QLQ-C30** QOL ↓ with UBM + for global QOL^+++^, physical, emotional, social, cognitive and role functioning^+++^, and pain and fatigue symptoms^+++^
	*160*	**–**	**–**	**–**		
Fu, 2022 [136]	**345**	**250**	**215**	**–**	**Lymphatic pain** BCLE-SEI questionnaire (Part I) score	**SF-36** QOL ↓with UBM + only compared to no symptoms^+^ or fatigue only^+++^. QOL ↓ with UBM + with fatigue compared to no symptoms ^+++^ or fatigue only^+++^ for overall health
	*215*	*140*	*160*	**–**		
	*139*	*75*	*90*	**–**		
Gong, 2020* [84]	*560*	*319*	*356*	*325*	**PMPS** Ipsilateral chest, axilla, shoulder, or arm pain > 3-monhs post-Sx	**EORTC QLQ-C30** ** + BR23** QOL ↓ with UBM + for global QOL^+^, physical function^+^, role function^+++^ and social function^+^. UBM + ↔ UBM − in emotional function and cognitive function
	*1423*	*830*	*850*	*882*		
Hamood, 2018* [83]	*303*	*249*	*181*	*249*	**Chronic pain** Pain presence/ severity rating (0**–**10)	**SF-36** QOL ↓ with UBM + for all SF-36 subscales^+++^
	*100*	*72*	*41*	*80*		
Hau, 2013 [64]	**428**	**22**	**82**	**165**	**Arm symptoms** Self-reported arm swelling, pain, limitation in arm movement, loss of feeling in fingers	**EORTC QLQ-C30** Global QOL ↓ with moderate or severe arm swelling^++^, arm pain^+++^, limitation of movement^++^, loss of feeling in fingers^+^
Hayes, 2022^ [137]	**2442**	**1499**	**1768**	**–**	**Upper-body symptoms** DASH and FACT-B + 4 (Arm symptom subscale) questionnaires	**FACT-B + 4** QOL ↓ with UBM + for total FACT-G^+++^, total FACT-B + 4^+++^, FACT-TOI^+++^, and arm symptoms subscale^+++^
Heiney, 2007* [65]	*120*	*66*	**–**	**–**	**LE** Self-reported hand swelling	**QOL-BCV** QOL ↓ with UBM + overall^+^ and for physical^+++^ and social^+++^ subscales
	*414*	*213*	**–**	**–**		
Hickey, 2011 [138]	*18*	*10*	*9*	*10*	**Persistent post-surgical pain** Pain in the last two weeks, attributed to Sx	**SF-36** UMB + ↔ UBM − in all subscales Trend towards ↓with UBM + for physical functioning (*p* = 0.055)
	*24*	*9*	*14*	*14*		
Hormes, 2010* [66]	**–**	**–**	**–**	**–**	**LE** < 10% interlimb volume difference or previous LE Dx	**SF-36:** QOL ↓ with UBM + for physical functioning^+++^, role functioning physical^+++^, role functioning emotional^+++^, social functioning^+++^, bodily pain^+++^, mental health^+++^, energy/fatigue^+++^, and general health perceptions^+++^
Jariwala, 2022* [92]	**212**	**155**	**187**	**91**	**Arm and shoulder problems** Kwan’s Arm Problem Scale score ≥ 21.5	**SF-36** QOL ↓ with UBM + for physical functioning^+^, physical role functioning^+^, bodily pain^+^, general health^+^, energy/fatigue^+^, and physical component score^++^
	**–**	**–**	**–**	**–**		
	**–**	**–**	**–**	**–**		
Jørgensen, 2021* [139]	**1067**	**929**	**738**	**862**	**LE** Clinical LE diagnosis	**SF-36** **LYMPH-ICF** QOL ↓ with UBM + for SF-36 total^+++^, physical role functioning^+++^, energy/fatigue, mental health, social role functioning, bodily pain, general health perceptions and all LYMPH-ICF subscales^+++^
	*244*	*230*	*204*	*198*		
	*823*	*699*	*534*	*664*		
Kaur, 2017* [85]	**210**	**–**	**–**	**–**	**Chronic post–mastectomy pain** VAS pain intensity > 3/10	**FACT-B** **FACT-G** QOL ↓ with UBM + for physical wellbeing^++^, emotional wellbeing^+++^, functional wellbeing^+^, the BC subscale^+++^, trial outcome index^+++^, total FACT-B^+++^, and total FACT-G^+++^ scores
	*319*	**–**	**–**	**–**		
	*47*	*40*	*43*	**–**		
Kibar, 2017* [140]	**201**	**184**	**95**	**–**	**Upper-extremity impairment** VAS pain > 3/10; Shoulder ROM > 20° below norm; Shoulder MMT < 4 MRC scale, any direction; Heaviness or numbness	**SF-36** QOL ↓ with UBM + for mental^+^ and physical^+^ component scores
	*107*	*70*	*95*	**–**		
	*94*	*46*	*80*	**–**		
Koca, 2020* [141]	**67**	**–**	**–**	**–**	**LE** Interlimb circumference difference	**WHOQOL-BREF** Difference exists between UBM + (LE, no symptoms), UBM + (LE symptoms), and UBM − groups for physical ^+^, psychological^+^, social ^+^, and environmental^+^ health subscales and total WHOQOL-BREF score^+^
	*15*	**–**	**–**	**–**		
	*51*	**–**	**–**	**–**		
Koehler, 2020 [63]	**748**	**525**	**490**	**507**	**LE** Self-report	**LYMPH-ICF UL** QOL ↓ with UBM + for all LYMPH-ICF subscales^+++^
	*290*	*208*	*222*	*204*		
	*458*	*317*	*268*	*303*		
Kwan, 2002 [142]	**–**	**–**	**–**	**–**	**Arm pain, stiffness, swelling, numbness, or LE** Self-reported symptoms or interlimb volume difference ≥ 200 ml	**EORTC QLQ-C30** Difference exists between UBM + (LE grade 1), UMB + (LE grade 2), and UBM − groups for physical functioning^++^, social functioning^++^, pain symptoms^+++^
	**–**	**–**	**–**	**–**		
Langford, 2015* [143]	*158*	*106*	*44*	**–**	**Post-surgical breast pain** Self-reported pain in affected breast	**QOL-PV** QOL ↓ with persisting UBM + for total QOL, physical wellbeing^+++^, psychological wellbeing^+++^, and social wellbeing^+++^
	*122*	*70*	*39*	*-*		
Lee, 2012* [144]	*58*	*17*	*49*	*54*	**LE** Arm circumference ≥ 2 cm greater than contralateral side	**SF-36** UBM + ↔ UBM − for all SF-36 subscales
	*38*	*14*	*28*	*32*		
Lopez-Penha, 2014* [67]	*26*	*19*	*18*	*20*	**LE** Interlimb volume difference > 200 ml	**EORTC QLQ-C30** ** + BR23** QOL ↓ with UBM + for physical functioning^++^, role functioning^++^, social functioning^++^, breast symptoms^+^ and arm symptoms^++^
	*119*	*90*	*44*	*58*		
Macdonald, 2005* [86]	**–**	**–**	**–**	**–**	**PMPS** Neuropathic chest wall, axilla, or arm pain on side of Sx > 3-months	**SF-36** QOL ↓ with UBM + for physical functioning^++^, role functioning physical^+^, bodily pain^+++^, general health perceptions^++^, energy/fatigue^+++^, social functioning^++^, and mental health^++^
	*59*	**–**	**–**	**–**		
	*54*	**–**	**–**	**–**		
Mak, 2009* [68]	*101*	**–**	**–**	**–**	**LE** Arm circumference ≥ 1.5 cm greater than contralateral side	**FACT-B + 4** QOL ↓ with UBM + for physical wellbeing^++^, social/family wellbeing^+^, functional wellbeing^++^, BC subscale^+++^, arm symptom subscale^+++^, and total FACT-B + 4 score^+++^
	*101*	**–**	**–**	**–**		
Mandelblatt, 2002* [145]	**571**	**300**	**-**	**–**	**Difficulties with arm functioning** Self-reported swelling, loss of arm movement, or limitation of use of hands/fingers on side of Sx	**SF-12** UBM + ↔ UBM − for mental and physical component scores
	**–**	**–**	**–**	**–**		
	**–**	**–**	**–**	**–**		
Meijuan, 2013* [87]	**220**	**6**	**184**	**-**	**PMPS** Neuropathic chest wall, axilla, or arm pain on side of Sx > 3-months	**SF-36:** QOL ↓ with UBM + for role functioning physical^+^, bodily pain^+^, energy/fatigue^+^, role functioning emotional^+^, mental health^+^, and general health perceptions^+^
	*61*	*1*	*51*	*-*		
	*163*	*5*	*133*	*-*		
Mülkoğlu, 2021 [146]	*25*	**–**	**–**	**–**	**LE** Interlimb circumference difference > 2 cm; Interlimb volume difference > 200 mL	**EORTC QLQ-C30** QOL ↓ with UBM + for physical functioning^+^
	*20*	**–**	**–**	**–**		
Nesvold, 2011* [147]	*80*	*80*	*67*	*59*	**Arm/shoulder problems** ≥ 2 of: Contralateral difference in shoulder ROM ≥ 25°; Contralateral difference in arm volume ≥ 10% or circumference ≥ 2 cm; Kwans Arm Problem Scale score ≥ 21.5	**SF-36** QOL ↓ with UBM + for physical functioning^+++^, physical role functioning^+++^, bodily pain^+++^, general health^+++^, energy/fatigue^+++^, social functioning^+++^, emotional role functioning^+++^, mental health^+++^, and physical component score^+++^
	*175*	*175*	*138*	*134*		
Neuner, 2014 [79]	**3083**	**1568**	**560**	**1840**	**LE** Reported LE Dx or hand or arm swelling on surgical side	**SF-12** UBM + predicts ↓ physical component score (− 9.5%) and ↓mental component score (− 5.2%) at 3 time points 2.5**–**5 years post-Dx (combined)
Oliveri, 2008* [148]	**245**	−	−	−	**LE** Hand or arm swelling from “LE and Pain Questionnaire” [149]	**SF-36** UBM + ↔ UBM − for physical component score and mental component score
	*75*	−	−	−		
	*170*	−	−	−		
Pinto, 2013* [100]	*50*	−	−	−	**LE** Stage I or II LE (International Society of Lymphology staging system)	**SF-12** UBM + ↔ UBM − for physical component score and mental component score. Trend towards ↓ mental component score with UBM + (*p* = 0.066)
	*50*	−	−	−		
Popovic-Petrovic, 2018* [150]	*34*	−	−	−	**LE** Clinical Dx via interlimb circumference difference	**FACT-B + 4** UBM + ↔ UBM − for all FACT-B + 4 subscales
	*30*	−	−	−		
Pyszel, 2006 [69]	−	−	−	−	**LE** Self-report	**EORTC QLQ-C30** ** + BR23** QOL ↓ with UBM + for all EORTC QLQ-C30 and functional subscales. Symptom scores ↑ with UBM + , excl. appetite loss QOL ↓ with UBM + for EORTC QLQ-BR23 future perspectives^+^. Symptom scores ↑ with UBM + for breast^+++^ and arm^+++^ symptom subscales
	−	−	−	−		
Recchia, 2005* [151]	*15*	−	−	−	**Pain** McGill pain questionnaire	**FACT-B + 4** QOL ↓ with UBM + for physical wellbeing^+++^, functional wellbeing^+^, and emotional wellbeing^+^, BC subscale^+^, arm symptom subscale^+^, and total FACT-B + 4^+++^ scores
	*15*	−	−	−		
Ridner, 2005* [70]	**128**	**66**	**83**	−	**LE** LE index ratio ≥ 1.139 via bioelectrical impedance	**FACT-B + 4** QOL ↓ with UBM + for total FACT-B score^++^
	*64*	*29*	*40*	−		
	*64*	*37*	*43*	−		
Round, 2006 [71]	**287**	−	−	−	**LE** Self-reported arm swelling	**FACT-B + 4** QOL ↓ with UBM + for total FACT-B + 4 score^++^
Speck, 2010* [152]	*112*	*91*	*92*	−	**LE** Previous clinical Dx of LE or interlimb volume difference ≥ 10%; pitting oedema; swelling on inspection	**SF-36** UBM + ↔ UBM − for physical and mental component scores in Tx and CG
	*118*	*90*	*84*	−		
Sürmeli, 2019* [153]	*27*	−	−	−	**LE** -	**EORTC QLQ-C30** QOL ↓ with UBM + for global QOL^+++^, physical functioning^+++^, cognitive functioning^+++^, and social functioning^+++^, role functioning^+^, emotional functioning^++^, and symptom score (composite)^++^
	*29*	−	−	−		
Tan, 2023 [154]	**210**	**51**	**8**	−	**Pain** VAS ≥ 3/10 or “Yes” to BPI pain impact items	**EQ-5D-3L** ↓ QOL associated with UBM + for general health status^+++^
	*135*	*31*	*5*	−		
	*75*	*20*	*3*	−		
Togawa, 2021* [72]	**499**	**327**	**239**	**321**	**LE** Self-reported persistent swelling on operated side	**SF-36** QOL ↓ with any UBM + for physical component score^+++^, physical role functioning^+++^, and bodily pain ^+++^. ↓ QOL with UMB + (LE symptomatic) for physical functioning^+++^ and general health^+++^. ↓ QOL with UMB + (LE asymptomatic) for social functioning^+++^
	*137*	*85*	*80*	*89*		
	*362*	*242*	*159*	*232*		
Vassard, 2010 [102]	*125*	*70*	*51*	−	**LE** Self-reported swelling in arms, onset after Sx	**EORTC QLQ-C30** UBM + ↔ UBM − for global QOL subscale. Trend towards ↓ QOL for UBM + (*p* = 0.08)
	*508*	*58*	*40*	−		
Velanovich, 1999 [73]	−	−	−	−	**LE** Interlimb circumference difference > 1 cm	**SF-36** QOL ↓ with UBM + for role functioning emotional^+^. Trend towards ↑ bodily pain for UBM (*p* = 0.08) subscales
	−	−	−	−		
Wilson, 2005* [74]	*32*	−	−	−	**LE** Previous Dx of/referral to receive Tx for LE	**SF-36** QOL ↓ with UBM + for all SF-36 subscales^++^ (adjusted) excl. mental health
	*78*	−	−	−		
Young-Afat, 2019* [75]	**836**	**836**	**137**	**656**	**Breast oedema** Breast swelling rated “quite a bit” or “very much” on EORTC QLQ-BR23	**EORTC QLQ-C30** QOL ↓ with UBM + for global health status^+^, physical functioning^+^, and body image^+^ subscales^+^
	*33*	*47*	*26*	*40*		
	−	−	−	−		
Yusof, 2021a* [76]	**113**	**92**	**86**	**86**	**LE** Self-report and interlimb circumference difference ≥ 1.5 cm at any two points on the arm	**FACT-B** **FACT-G** QOL ↓ with UBM + for physical wellbeing^+++^, functional wellbeing^++^, breast cancer subscale^+^, trial outcome index^+++^, total FACT-B score^++^, and total FACT-G score^+^
	−	−	−	−		
	−	−	−	−		
Yusof, 2021b* [77]	**160**	−	−	−	**LE** Self-report and Interlimb circumference difference ≥ 1.5 cm at any two points on the arm	**FACT-B** QOL ↓ with UBM + for total FACT-B score^+++^
	*33*	*24*	*25*	*27*		
	*127*	*94*	*105*	*82*		
*Zhao, 2020 *[78]	*155*	*61*	*152*	*41*	**LE** Interlimb circumference difference ≥ 2 cm	**LYMPH-ICF-UL** QOL ↓ with UBM + for all Lymph-ICF-UL subscales^+++^
	*90*	*22*	*90*	*5*		

Breast Cancer (BC); Breast Cancer and Lymphedema Symptom Experience Index (BCLE-SEI); Breast (Br); Axilla (Ax); Treatment (Tx); Diagnosis (Dx); Surgery (Sx); Radiotherapy (RT); Chemotherapy (CT); Endocrine therapy (ET); Infiltrating Ductal Carcinoma (IDC); Infiltrating Lobular Carcinoma (ILC); Standard deviation (sd); Sum of squares (SS); Med (Median); Interquartile range (IQR); Cancer And Leukemia Group B(CALG-B); Lymphedema (LE); Post-Mastectomy Pain Syndrome (PMPS). Assessments/questionnaires: World Health Organisation Quality of Life questionnaire, Brief (WHOQOL-BREF); European Organisation for the Research and Treatment of Cancer Quality of Life Questionnaire, Core 30 (EORTC QLQ-C30); European Organisation for the Research and Treatment of Cancer Quality of Life Questionnaire, Breast cancer module (EORTC QLQ-BR23); European Quality of Life 5 Dimensions 3 Level Version (EQ-5D-3L); Functional Assessment of Cancer Therapy, Breast (with arm symptoms subscale)(FACT-B + 4); Functional Assessment of Cancer Therapy, General (FACT-G); Functional Assessment of Cancer Therapy, Breast—Trial Outcome Index (FACT-B-TOI); Short form 12 (SF-12); Short form 36 (SF-36); Douleur neuropathique-4 questionnaire (DN-4); Manual Muscle Test (MMT); Medical Research Council (MRC); Psychological General Wellbeing index (PGWB); Disabilities of the Arm, Shoulder, and Hand questionnaire (DASH [155]; 20-item Quality of life questionnaire; Psychological General Well-Being index (PGWB); Lymphedema Functioning Disability and Health questionnaire for upper-limb lymphedema (LYMPH-ICF UL); The Quality of Life scale – Patient version (QOL-PV); The Quality of Life scale – Breast Cancer version (QOL-BCV)

^*^Study included in meta-analysis

^Treatment/participant characteristics recorded at baseline, prior to UBM or QOL assessment

–Data not reported/presented

^+^ = p < 0.05; ^++^p =  < 0.01; ^+++^p ≤ 0.001

Fifty-seven studies reported the methods used to determine the presence of UBM, and these were self-report/questionnaire responses (*n* = 34), objective measures (*n* = 14), or a combination of the two (*n* = 9). One study did not describe the method used to categorise participants as lymphoedema positive or negative [38]. Questionnaires used alone or in combination to assess UBM included the McGill Pain Questionnaire [39] (*n* = 3), Brief Pain Inventory [40] (*n* = 2), Disabilities of the Arm, Shoulder and Hand questionnaire [41] (*n* = 2), Visual Analogue Scale [42] (*n* = 4), lymphoedema and pain questionnaire [43] (*n* = 1), Douleur Neuropathique-4 questionnaire [44] (*n* = 1), unspecified/custom UBM/Lymphoedema questionnaire (*n* = 5), The Breast Cancer and Lymphedema Symptom Experience Index (BCLE-SEI) [45] (*n* = 1), Functional Assessment of Cancer Therapy, Breast-Arm Symptom Subscale [46] (*n* = 1), or the “breast swelling” item on the EORTC QLQ-BR23 questionnaire [47] (*n* = 1). Objective measures used to identify lymphoedema were upper-limb circumference (*n* = 11), perometry (*n* = 1), bioelectrical impedance (*n* = 1), and volumetric displacement (*n* = 1). Impaired shoulder ROM was quantified using goniometry (*n* = 3).

QOL was assessed using the following tools: Medical Outcomes Study – Short form 36 (SF-36) [48] (*n* = 19); European Organisation for Research and Treatment of Cancer, Quality of life Questionnaire – Core (EORTC QLQ-C30) [49] (*n* = 13) and/or breast module (EORTC QLQ-BR23) [47] (*n* = 4); Functional Assessment of Cancer Therapy, Breast (FACT-B) [46] (*n* = 5) with arm symptoms subscale (FACT-B + 4) [50] (*n* = 9); Medical Outcomes Study – Short form 12 [51] (*n* = 4); Lymphedema Functioning Disability and health questionnaire for upper-limb lymphedema (LYMPH-ICF-UL) [52] (*n* = 3); World Health Organisation Quality of Life Questionnaire, brief (WHOQOL-BREF) [53] (*n* = 2); 20-item Quality of life questionnaire [54] (*n* = 1); Psychological General Well-Being index (PGWB) [55] (*n* = 1); The Quality of Life scale – Patient version [56] (*n* = 1); The Quality of Life scale – Breast Cancer version [57] (*n* = 1), and the European Quality of Life 5 Dimensions 3 Level Version questionnaire (EQ-5D-3L) [58] (*n* = 1).

Statistically significant differences between UBM + and UBM − groups existed across several QOL domains. Groups with lymphoedema [14, 38, 59–79], pain [54, 64, 80–88], movement limitations [4, 64], upper-body disability [89], or a combination of UBM types [16, 18, 90–93] reported poorer QOL than UBM − groups in at least one domain. Where QOL was not significantly different between groups [94–96], or no statistical analysis was presented [97] mean or median subscale scores tended to be lower in those with UBM compared to those without [94, 95, 98–102], particularly with respect to physical symptoms. Few studies reported trends towards superior QOL in UBM − groups, in terms of severity of arm symptoms [103] and physical wellbeing [18, 96, 99, 101], mental wellbeing [96], and global QOL, physical role, emotional role, cognitive functioning and social functioning [18].

## Primary analysis

**Fig. 2:**
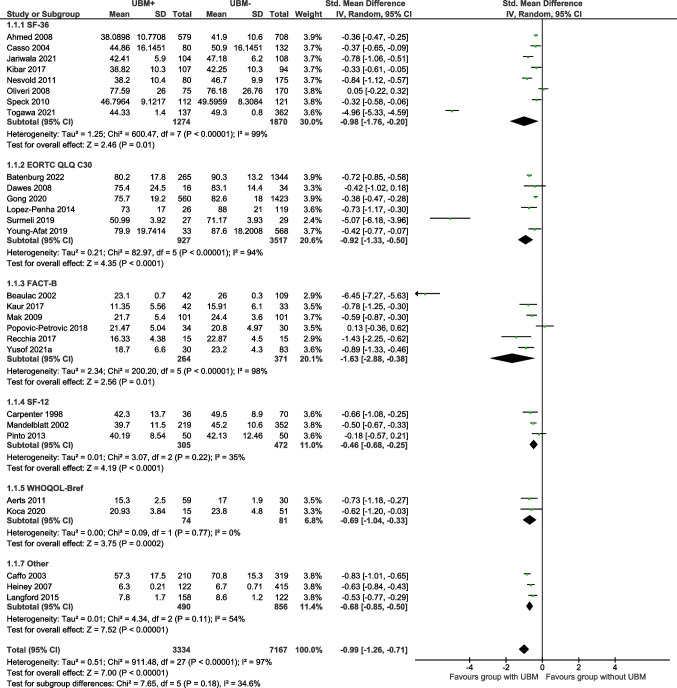
The effect of UBM on QOL (SMD): physical wellbeing

Physical wellbeing was reported in 28 studies using eight different QOL assessment tools. The relevant physical wellbeing, physical functioning, or physical component scores from eight QOL assessment tools were included in the meta-analysis. Overall, physical wellbeing was significantly poorer in the UBM + group, with UBM exerting a large negative effect on scores in this domain across all questionnaires (SMD =  − 0.99; 95%CI =  − 1.26, − 0.71; Z = 7.00; df = 27; *p* < 0.00001) [Total (*n* = 10,501); UBM + (*n* = 3334); UBM − (*n* = 7167)] (Fig. 2).

**Fig. 3:**
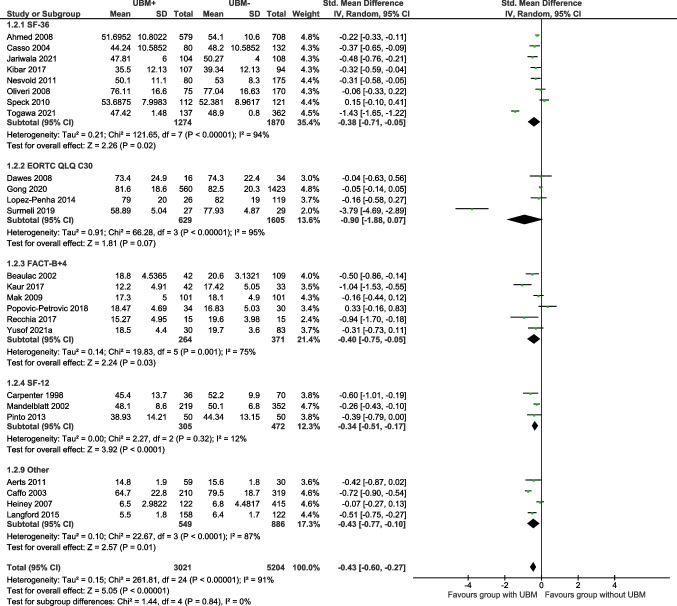
The effect of UBM on QOL (SMD): psychological/emotional wellbeing

Psychological/emotional wellbeing was reported in 25 studies using eight QOL assessment tools. Psychological/emotional wellbeing was significantly poorer in the UBM + group with a moderate effect size (SMD =  − 0.43; 95%CI =  − 0.60, − 0.27; Z = 5.05; df = 24; *p* < 0.00001) [Total (*n* = 8225); UBM + (*n* = 3021); UBM − (* n* = 5204)] (Fig. 3). There was evidence to suggest a significant negative effect of UBM for psychological/emotional wellbeing measured using the SF-36 (*p* < 0.00001), FACT-B (*p* = 0.001), EORTC-QLQ C30 (*p* < 0.00001), and ‘other’ questionnaires (*p* < 0.0001). There was no between group differences in SF-12 questionnaire scores (*p* = 0.32).

**Fig. 4:**
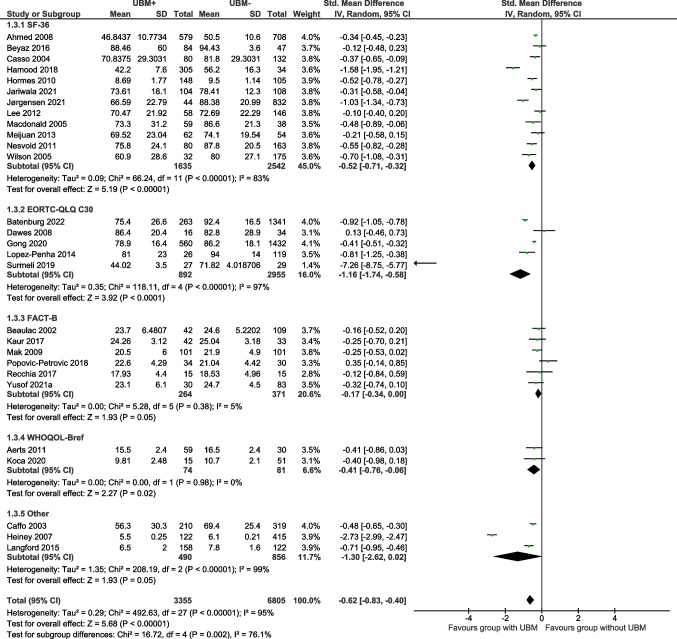
The effect of UBM on QOL (SMD): social wellbeing

Social wellbeing/function was reported in 28 studies using seven QOL assessment tools. Overall, social wellbeing/function was significantly poorer in the UBM + group, with a moderate to large effect size (SMD =  − 0.62; 95%CI =  − 0.83, − 0.40; Z = 5.68; df = 27; *p* < 0.00001) [Total (*n* = 10,160); UBM + (*n* = 3355); UBM − (* n* = 6805)] (Fig. 4). Moderate and large significant negative effects of UBM were observed in studies using the SF-36 (SMD =  − 0.52; 95%CI =  − 0.71, − 0.32; Z = 5.19; df = 11; *p* < 0.00001) and EORTC QLQ-C30 questionnaires, respectively (SMD =  − 1.16; 95%CI =  − 1.74, − 0.58; Z = 3.92; df = 4; *p* < 0.00001) and ‘other’ questionnaires (SMD =  − 1.30; 95%CI =  − 2.62, 0.02; Z = 1.93; df = 2; *p* < 0.00001). No significant differences were observed between groups for the FACT-B (*p* = 0.38) or WHOQOL-Bref (*p* = 0.98) questionnaires.

The sensitivity analysis (Online resource 1) showed that excluding studies which used objective measures of UBM had a minor impact on the magnitude, but not on the direction or significance of the effect of UBM on QOL. Including individuals with objective UBM (e.g. clinically diagnosed lymphoedema) in the analysis does not significantly diminish the size of the effect, irrespective of whether they experience adverse symptoms (e.g. discomfort) or not.

## Study quality

**Fig. 5:**
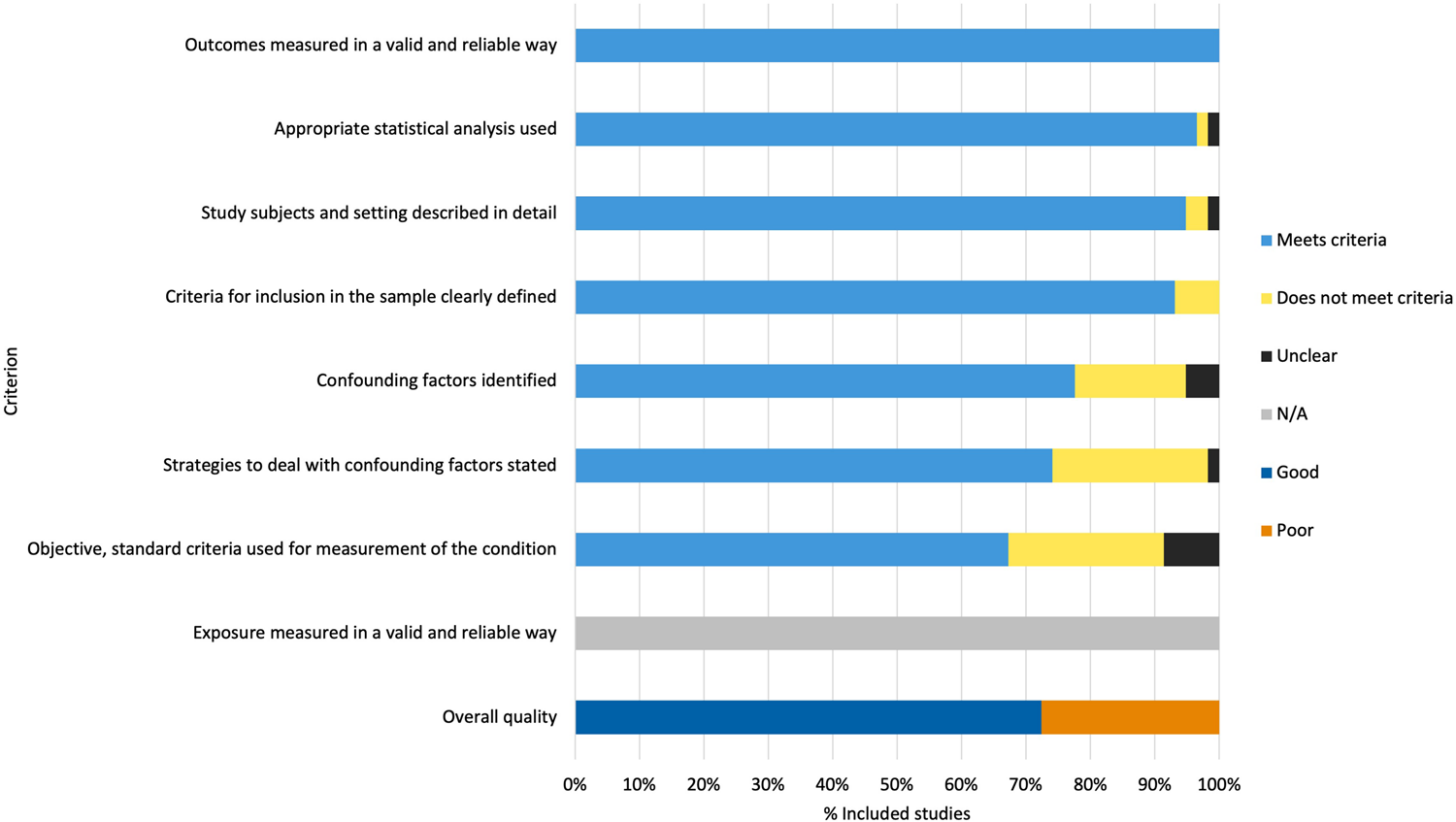
Quality of included studies: Joanna Briggs Institute checklist for analytical cross-sectional studies [30]

The results of the study quality assessment are summarised in Fig. 5 and presented in full in Online resource 1. Results are displayed as the proportion of included studies meeting each JBI checklist item. Of the 58 included studies, 72.4% were rated as good quality. Of those studies included in the meta-analysis, 71.8% were rated as good quality. Reasons for poor quality ratings included insufficient description of the study inclusion criteria and sample characteristics, failure to describe the criteria for the classification into UBM + and UBM − groups, lack of appropriate statistical analysis, and inadequate controlling of confounding variables.

## Evaluation of publication bias

Funnel plots for each of the primary analyses showed asymmetrical distribution of studies either side of the main effect (Online resource 1) inferring the presence of publication bias, such as failure to publish small studies with insignificant effects estimates. This may have contributed to an overestimation of the effect of UBM on wellbeing scores.

## Exploratory analyses

In the exploratory analyses, studies were grouped according to QOL questionnaire. Domain scores were compared between UBM + and UBM − groups. Differences in scores were given clinical context by way of comparison to predetermined MID or MCID thresholds [27, 35], available for some widely used and validated questionnaires including the SF-36, SF-12, and EORTC QLQ-C30 [35, 104, 105]. UBM demonstrated a negative effect of clinically important magnitude, across all subscales of the SF-36 and SF-12 questionnaires. Furthermore, there was a significant negative effect on physical and social health scores on the WHOQOL-BREF questionnaire due to UBM. No difference existed between UBM + and UBM − groups for EORTC QLQ-C30 emotional or cognitive functioning, EORTC QLQ-BR23 body image, sexual function, sexual enjoyment, arm symptoms, or future perspectives, or FACT-B + 4 social/family wellbeing. Findings from the exploratory analysis are summarised in Table 2. Forest plots from each analysis are available in the supplementary material (Online resource 1).

**Table 2:** Summary of exploratory findings: The effect of upper-body morbidity on quality of life according to questionnaire

	Questionnaire subscales	
Questionnaire	Negative effect due to UBM	No effect due to UBM
SF-36	Physical wellbeing^‡^	^†^
	Physical role functioning^‡^	
	Emotional role functioning^‡^	
	Energy/fatigue^‡^	
	Mental health^‡^	
	Social function^‡^	
	Bodily pain^‡^	
	General health^‡^	
SF-12	Physical component score^‡^	^†^
	Mental component score^‡^	
EORTC QLQ-C30	Global health status^‡^	Emotional functioning
	Physical functioning^‡^	Cognitive functioning
	Role functioning^‡^	
	Social functioning^‡^	
EORTC QLQ-BR23	Breast symptoms	Body image
		Sexual function
		Sexual enjoyment
		Arm symptoms
		Future perspectives
FACT-B + 4	Total FACT-B^‡^	Social/family wellbeing
	Total FACT-B + 4	
	Physical wellbeing	
	Emotional wellbeing	
	Functional wellbeing	
	Breast cancer subscale^‡^	
	Arm symptom subscale	
WHOQOL-BREF	Physical health	Environmental health
	Social relationships	General health

World Health Organisation Quality of Life questionnaire, Brief (WHOQOL-BREF); European Organisation for the Research and Treatment of Cancer Quality of Life Questionnaire, Core 30 (EORTC QLQ-C30); European Organisation for the Research and Treatment of Cancer Quality of Life Questionnaire, Breast cancer module (EORTC QLQ-BR23) Functional Assessment of Cancer Therapy, Breast (with arm symptoms subscale) (FACT-B + 4); Short form 12 (SF-12); Short form 36 (SF-36)

^†^No applicable subscales

^‡^Exceeds MCID/MID for questionnaire subscale

## Discussion

The aim of the present study was to evaluate the effect of breast cancer treatment-related UBM on QOL. The primary analyses demonstrated that physical, psychological/emotional, and social aspects of QOL were negatively impacted by the presence of UBM after treatment. However, the degree to which each of these domains was affected, varied. Difference in QOL was most substantial in terms of physical wellbeing and function, as would be expected given the presence of physical upper-body symptoms and limitations differentiating the two groups. Detriment to physical QOL domains has previously been attributed to the difficulty UBM introduces to performing routine tasks such as cooking, cleaning, dressing/grooming and driving [106, 107]. The present analysis also revealed that beyond being a source of physical morbidity, UBM is associated with impairment to social function and psychological wellbeing. This echoes findings from studies that have identified UBM as a source of distress and psychological burden [107]. Experiencing UBM may magnify the discrepancy between one’s pre- and post-cancer capabilities — for example, the inability to perform usual roles within home, social and work context — explaining to some extent, why UBM contributes to impaired psychological and social wellbeing [14, 16, 24, 107, 108].

The review included studies that reported QOL after breast cancer using a variety of general or cancer-specific multidimensional QOL tools, warranting exploratory analyses with studies grouped according to questionnaire. These analyses also revealed substantial impairment across several domains of QOL due to UBM. However, the direction and size of the effect of UBM on corresponding subscales of different questionnaires varied (Table 2), and in some instances, contrasted findings from the primary analysis. For example, UBM had no effect on social functioning or social/family wellbeing subscales of the EORTC QLQ-C30 and FACT-B questionnaires, respectively, yet demonstrated a negative effect on SF-36 social function and WHOQOL-BREF social relationships subscales. Effects were also inconsistent between questionnaires for emotional functioning, general health/global QOL, and breast/arm symptoms subscales. The variable impact of UBM on QOL according to questionnaire may be accounted for by disparities in the number of studies included in each exploratory analysis. Other factors including sample demographics, treatment regime, and UBM type, duration, and severity, have been identified as moderators of the effect of UBM on QOL and may have contributed to the variable effects observed [109–111].

It is also worth considering the potential impact of questionnaire selection, on assessing QOL across the cancer continuum [112, 113]. Cancer-specific questionnaires, designed to assess QOL during active treatment when patients experience acute treatment side effects, new psychosocial stressors, and fears about the future, may not contain items of relevance to longer term cancer survivors [114–116]. Conversely, generic assessment tools fail to capture the presence of specific cancer/treatment-related effects and their impact on QOL. Selecting a tool with coverage of concerns relevant to a person’s stage on the cancer continuum is paramount to accurate and informative QOL assessment [112]. To improve detection of impaired QOL going forward, administration of a combination of cancer-specific and generic questionnaires may be indicated.

This review represents a comprehensive study of the literature describing multiple types of UBM and their relationship to QOL. It is the first to produce a meta-analysis quantifying the overall effect of UBM on key QOL domains, and the effect of UBM on QOL scores from individual questionnaires.

## Study limitations

There are limitations to consider, the first related to the types of UBM reported and methods used to categorise individuals as UBM + or UBM − . The majority of included studies compared individuals with or without lymphoedema. As a prevalent type of UBM after breast cancer there is merit in assessing the impact of lymphoedema on QOL, but findings of these meta-analyses may not reflect the impact of other types of UBM on QOL. Furthermore, the dichotomous classification of UBM represents a limitation to appreciating the complexities of its effect on QOL. For example, the influence of UBM severity, UBM duration/time since treatment, and UBM type is obscured by categorising individuals into discrete UBM + and UBM − groups. A comprehensive meta-analysis in which UBM is further stratified according to type and severity and accounts for time since treatment may address this limitation. However, this may not be feasible given the heterogeneity of currently available data, and the potential co-occurrence of multiple types of UBM (e.g. pain associated with lymphoedema).

Second, as QOL is a multidimensional construct, this review sought to determine the differential impact of UBM on multiple life domains. As such, only studies that employed multidimensional QOL assessment tools were included. Studies using questionnaires to assess components of wellbeing such as anxiety and depression severity, functional impairment, or body image, were excluded. Viewed alongside this review these measures may add richness to the understanding of breast cancer survivor experiences of UBM after treatment.

Finally, the risk of bias and potential overestimation of the observed effect should be addressed. Funnel plots generated for the primary analysis were asymmetrical, inferring risk of publication bias [34]. Additional sources of bias may have included the poor reporting and methodological quality, evident in the ‘poor’ quality rating given to ~ 30% of studies, and the high level of heterogeneity between studies in terms of time since treatment, UBM type, and criteria for assignment to UBM + and UBM − groups existed between studies.

## Clinical implications

Whilst this review does not provide evidence endorsing strategies to prevent or manage UBM, the findings justify efforts taken to minimise the presence and impact of UBM to preserve QOL. In the literature to date, examples of such strategies include the selection of minimally invasive procedures to minimise the risk of developing UBM [117–121]; implementation of “Prehabilitation” to improve physical and psychological condition prior to initiating breast cancer treatment and promote superior treatment outcomes [122–127]; and the implementation of “Rehabilitation”, such as physical therapy/exercise or activities to promote recovery to pre-treatment physical capacity and QOL [126–130]. Based on the findings of this review, there is merit in implementing UBM prevention and management strategies that address multiple aspects of wellbeing, in order to effectively minimise impairment to overall QOL [7, 131].

## Conclusions

Individuals with breast cancer-related UBM that persists beyond primary treatment, report significantly poorer QOL than individuals without UBM. While the most substantial negative effects were observed in physical wellbeing and functioning domains, evidence showed that several domains of QOL are subject to impairment in groups with UBM. There is merit in assessing impairment due to UBM using relevant, multidimensional QOL assessment tools. The pursuit of strategies to prevent and manage UBM is warranted, to minimise its impact on physical, psychological, and social wellbeing across the cancer continuum.

## Supplementary Information

Below is the link to the electronic supplementary material.

Supplementary file1 (DOCX 1752 KB)

## Data Availability

Template data collection forms and extracted data used for analysis are available upon reasonable request to the corresponding author.

## Declarations

### Disclaimer

The authors wish to note that the submitted manuscript is longer than the average, as outlined in the *“Journal of Cancer Survivorship”* submission guidelines. This is due to the inclusion of sizeable tables and figures, which demonstrate rigorous adherence to PRISMA guidelines and the scale/completeness of the systematic review and meta-analysis. The authors acknowledge that the *“Journal of Cancer Survivorship”* values high quality, transparent reporting, and welcome feedback to optimise this review for its readership.
