# Supplementary material for: Evaluating the effect of upper-body morbidity on quality of life following primary breast cancer treatment: a systematic review and meta-analysis
Source: J Cancer Surviv. 2023 May 18;18(5):1517–47. doi: 10.1007/s11764-023-01395-0 (PMC11424680; doi:10.1007/s11764-023-01395-0)
Supplement: Supplementary file 1 — Supplementary file1 (DOCX 1752 KB) [file 11764_2023_1395_MOESM1_ESM.docx]

*Journal of Cancer Survivorship*

**Evaluating the effect of upper-body morbidity on quality of life following primary breast cancer treatment: A systematic review and meta-analysis.**

Eliza R. Maconald^1^, Nadia M.L. Amorim^2^, Amanda D. Hagstrom^1^, Katarina Markovic^1^, David Simar^1^, Rachel E. Ward^1^ (Co-senior author), and Briana K. Clifford^1,3^ (Co-senior author).

*^1^ UNSW, Sydney, School of Health Sciences, Department of Exercise Physiology, Sydney, Australia.*

*^2^ Centre for Inflammation, Centenary Institute and University of Technology Sydney, Faculty of Science, School of Life Sciences, Sydney, NSW, Australia.*

*^3^ The University of Queensland (UQ), School of Nursing, Midwifery and Social Work, Brisbane, Australia.*

ESM 1 Study search strategy

| **Supplementary Table 1.** Systematic review search strategy | |
| --- | --- |
| **Database** | **Search terms** |
| **CINAHL**  (n=1071) | (( DE “BREAST cancer” OR DE “BREAST cancer in men” ) OR ((breast N4 cancer*) or breast carcinoma* or breast neoplasm* or breast tumo#r* or mammary carcinoma* ) OR breast cancer N3 survivor* )) AND (DE “QUALITY of life” OR ( “quality of life” or QOL or “health-related quality of life” or HRQOL ) OR ("SF-36" or "RAND-36" or "EORTC-QLQ" or "FACT-B") AND ( (DE “ARM”) OR (DE “CHEST (Anatomy)” OR (DE “BREAST”) OR (DE “SHOULDER”) OR (DE “PAIN”) OR (DE “EDEMA”) OR (DE “RANGE of motion of joints”) OR (DE “MUSCLE strength”) ) OR (((shoulder or arm or upper-extremit*) N4 (morbidity or dysfunction* or symptom* or problem* or pain or “range of motion”)) or (mastectomy N5 pain) or lymph#edema or “DASH”) |
| **Embase**  (n=9908)  **Emcare**  (n=2986) | 1. exp breast cancer/ 2. ((breast adj5 cancer*) or breast carcinoma* or breast tumo?r* or mammary carcinoma*) or (breast cancer ADJ4 survivor*) 3. 1 or 2 4. “quality of life”/ 5. “quality of life” or QOL or “health-related quality of life” or HRQOL or (“SF-36” or “RAND-36” or “EORTC-QLQ” or “FACT-B”) 6. 4 OR 5 7. exp “upper limb”/ or “breast”/ or “thorax”/ or exp “pain”/ or “lymphedema”/ or “breast cancer-related lymphedema”/ or “muscle strength”/ or “range of motion”/ 8, (((shoulder or arm or upper-extremit*) adj5 (morbidity or dysfunction* or symptom* or problem* or pain or “range of motion”)) or (mastectomy adj6 pain) or Lymph?edema or “DASH”) 9. 7 OR 8 10. 3 AND 6 AND 9 |
| **PubMed/Medline**  (n=2705) | (((breast neoplasms[MeSH Terms]) OR (breast cancer* OR breast carcinoma* OR breast tumour* OR breast tumor* OR Mammary carcinoma* or breast cancer survivor*)) AND ((quality of life[MeSH Terms:noexp]) OR ("quality of life" or "QOL" or "health-related quality of life" or "HRQOL") OR ("SF-36" or "RAND-36" or "EORTC-QLQ" or "FACT-B")) AND ((Upper extremity[MeSH Terms]) OR (breast[MeSH Terms]) OR (thorax[MeSH Terms:noexp]) OR (pain[MeSH Terms]) OR (lymphedema[MeSH Terms:noexp]) OR (breast cancer lymphedema[MeSH Terms]) OR (range of motion, articular[MeSH Terms]) OR (muscle strength[MeSH Terms]) OR (shoulder morbidity or shoulder dysfunction* or shoulder symptom* or shoulder problem* or shoulder pain* or "shoulder range of motion") OR (arm morbidity or arm dysfunction* or arm symptom* or arm problem* or arm pain* or (arm range of motion)) OR (upper extremit* morbidity or upper extremit* dysfunction* or upper extremit* symptom* or upper extremit* problem* or upper extremit* pain* or "upper extremity range of motion") OR mastectomy pain OR lymphedema OR lymphoedema OR "DASH")) |
| **PsycInfo**  (n=145) | (MAINSUBJECT.EXACT.EXPLODE("Breast Neoplasms") OR ((noft(breast n5 cancer*)) OR (noft(breast carcinoma*)) OR (noft(breast tumo?r*)) OR (noft(mammary carcinoma*)) noft(of) (noft("breast cancer n4 survivor*")))) AND ((MAINSUBJECT.EXACT.EXPLODE("Quality of Life") OR MAINSUBJECT.EXACT("Health Related Quality of Life")) OR ((noft("quality of life")) OR (noft(QOL)) OR (noft("health-related quality of life")) OR (noft(HRQL)) OR (noft("SF-36")) OR (noft("RAND-36")) OR (noft("EORTC-QLQ")) OR (noft("FACT-B")))) AND ((MAINSUBJECT.EXACT.EXPLODE("Thorax") OR MAINSUBJECT.EXACT.EXPLODE("Range of Motion") OR MAINSUBJECT.EXACT.EXPLODE("Pain") OR MAINSUBJECT.EXACT.EXPLODE("Edema") OR MAINSUBJECT.EXACT.EXPLODE("Physical Strength") OR MAINSUBJECT.EXACT.EXPLODE("Arm (Anatomy)")) OR (((noft(Shoulder)) OR (noft(arm)) OR (noft(upper extremit*))) noft(n5) ((noft(morbidity)) OR (noft(dysfunction*)) OR (noft(symptom*)) OR (noft(problem*)) OR (noft(pain)) OR (noft("range of motion"))) OR (noft(mastectomy n6 pain)) OR (noft(lymph?edema)) OR (noft("DASH")))) |
| **SPORTDiscus**  (n=105) | ( DE "BREAST cancer" OR DE "BREAST cancer in men" ) OR ( (breast N4 cancer*) or breast carcinoma* or breast neoplasm* or breast tumo#r* or mammary carcinoma* ) OR breast cancer N3 survivor* AND ( DE "QUALITY of life" ) OR ( "quality of life" or QOL or "health-related quality of life" or HRQOL ) OR ("SF-36" or "RAND-36" or "EORTC-QLQ" or "FACT-B") AND ( (DE "PAIN") OR (DE "EDEMA") OR (DE "RANGE of motion of joints") OR (DE "MUSCLE strength") ) OR ( (DE "ARM") OR (DE "CHEST (Anatomy)" OR (DE "BREAST") OR (DE "SHOULDER") ) OR (((shoulder or arm or upper-extremit*) N4 (morbidity or dysfunction* or symptom* or problem* or pain or “range of motion”)) or (mastectomy N5 pain) or lymph#edema or “DASH”) |


| **Supplementary Table 2.** Quality assessment: Joanna Briggs Institute Checklist for Analytical Cross-Sectional Studies | | | | | | | | | | | |
| --- | --- | --- | --- | --- | --- | --- | --- | --- | --- | --- | --- |
| **Author** | **Year** | **Criteria for inclusion in the sample clearly defined** | **Study subjects and setting described in detail** | **Exposure measured in a valid and reliable way** | **Objective, standard criteria used to measure the condition** | **Confounding factors identified** | **Strategies to deal with confounding factors stated** | **Outcomes measured in a valid and reliable way** | **Appropriate statistical analysis used** | **% Criteria met** | **Study quality** |
| Aerts (6) | 2011 | Y | Y | N/A | Y | Y | Y | Y | Y | 87.5 | **Good** |
| Ahmed (55) | 2008 | Y | Y | N/A | Y | Y | Y | Y | Y | 87.5 | **Good** |
| Batenburg (127) | 2002 | Y | Y | N/A | N | N | N | Y | N | 37.5 | **Poor** |
| Beaulac (56) | 2002 | Y | Y | N/A | Y | Y | Y | Y | Y | 87.5 | **Good** |
| Bell (76) | 2014 | Y | Y | N/A | N | Y | Y | Y | Y | 75 | **Good** |
| Beyaz (77) | 2016 | Y | Y | N/A | Y | N | N | Y | Y | 62.5 | **Poor** |
| Bulley (128) | 2013 | Y | Y | N/A | Y | Y | U | Y | Y | 75 | **Good** |
| Bundred(57) | 2020 | Y | Y | N/A | Y | Y | Y | Y | Y | 87.5 | **Good** |
| Caffo (51) | 2003 | Y | Y | N/A | Y | Y | Y | Y | Y | 87.5 | **Good** |
| Carpenter (78) | 1998 | Y | Y | N/A | Y | Y | N | Y | Y | 75 | **Good** |
| Casso (86) | 2004 | Y | Y | N/A | Y | Y | Y | Y | Y | 87.5 | **Good** |
| Chachaj (58) | 2010 | Y | Y | N/A | Y | Y | Y | Y | Y | 87.5 | **Good** |
| Dawes(90) | 2008 | Y | Y | N/A | Y | Y | Y | Y | Y | 87.5 | **Good** |
| DiSipio (85) | 2009 | Y | Y | N/A | Y | Y | Y | Y | Y | 87.5 | **Good** |
| Engel (87) | 2003 | Y | Y | N/A | N | Y | Y | Y | Y | 75 | **Good** |
| Fu (129) | 2022 | Y | Y | N/A | Y | Y | Y | Y | Y | 87.5 | **Good** |
| Gong (80) | 2020 | Y | Y | N/A | Y | Y | Y | Y | Y | 87.5 | **Good** |
| Hamood (130) | 2018 | Y | Y | N/A | Y | Y | Y | Y | Y | 87.5 | **Good** |
| Hau (60) | 2013 | Y | Y | N/A | N | Y | Y | Y | Y | 75 | **Good** |
| Hayes (5) | 2022 | Y | Y | N/A | Y | Y | Y | Y | Y | 87.5 | **Good** |
| Heiney (61) | 2007 | N | Y | N/A | N | Y | Y | Y | Y | 62.5 | **Poor** |
| Hickey (91) | 2011 | Y | Y | N/A | Y | N | N | Y | Y | 62.5 | **Poor** |
| Hormes (62) | 2010 | Y | N | N/A | Y | Y | Y | Y | Y | 75 | **Good** |
| Jariwala(88) | 2021 | Y | Y | N/A | Y | N | N | Y | Y | 62.5 | **Poor** |
| Jørgensen(13) | 2021 | Y | Y | N/A | U | Y | Y | Y | Y | 75 | **Good** |
| Kaur (81) | 2017 | Y | Y | N/A | Y | N | N | Y | Y | 62.5 | **Poor** |
| Kibar (89) | 2017 | Y | Y | N/A | Y | Y | Y | Y | Y | 87.5 | **Good** |
| Koca(131) | 2020 | Y | Y | N/A | Y | Y | Y | Y | Y | 87.5 | **Good** |
| Koehler(59) | 2020 | Y | U | N/A | N | N | N | Y | Y | 37.5 | **Poor** |
| Kwan (17) | 2002 | Y | N | N/A | N | Y | Y | Y | Y | 62.5 | **Poor** |
| Langford (132) | 2015 | Y | Y | N/A | Y | Y | Y | Y | Y | 87.5 | **Good** |
| Lee (93) | 2012 | Y | Y | N/A | Y | Y | Y | Y | Y | 87.5 | **Good** |
| LopezPenha (63) | 2016 | Y | Y | N/A | Y | Y | Y | Y | Y | 87.5 | **Good** |
| Macdonald (82) | 2005 | Y | Y | N/A | Y | Y | Y | Y | Y | 87.5 | **Good** |
| Mak (64) | 2009 | Y | Y | N/A | Y | U | N | Y | Y | 62.5 | **Poor** |
| Mandelblatt(92) | 2002 | Y | Y | N/A | U | Y | Y | Y | Y | 75 | **Good** |
| Meijuan(83) | 2013 | Y | Y | N/A | Y | Y | Y | Y | Y | 87.5 | **Good** |
| Mülkoğlu(133) | 2021 | Y | Y | N/A | Y | N | N | Y | Y | 62.5 | **Poor** |
| Nesvold(15) | 2011 | Y | Y | N/A | Y | Y | Y | Y | Y | 87.5 | **Good** |
| Neuner(75) | 2014 | Y | Y | N/A | N | Y | Y | Y | Y | 75 | **Good** |
| Oliveri (94) | 2008 | N | Y | N/A | N | N | N | Y | U | 25 | **Poor** |
| Pinto (95) | 2013 | Y | Y | N/A | Y | Y | Y | Y | Y | 87.5 | **Good** |
| Popovic-Petrovic (98) | 2018 | Y | Y | N/A | Y | Y | Y | Y | Y | 87.5 | **Good** |
| Pyszel (65) | 2006 | N | Y | N/A | N | Y | Y | Y | Y | 62.5 | **Poor** |
| Recchia (103) | 2017 | Y | Y | N/A | U | U | N | Y | Y | 50 | **Poor** |
| Ridner (66) | 2005 | Y | Y | N/A | Y | Y | Y | Y | Y | 87.5 | **Good** |
| Round (67) | 2006 | Y | Y | N/A | N | Y | Y | Y | Y | 75 | **Good** |
| Speck (96) | 2010 | Y | Y | N/A | Y | Y | Y | Y | Y | 87.5 | **Good** |
| Sürmeli (36) | 2019 | Y | Y | N/A | U | U | N | Y | Y | 50 | **Poor** |
| Tan (134) | 2023 | Y | Y | N/A | Y | Y | Y | Y | Y | 87.5 | **Good** |
| Togawa(68) | 2021 | Y | Y | N/A | N | Y | Y | Y | Y | 75 | **Good** |
| Vassard (97) | 2010 | Y | Y | N/A | N | Y | Y | Y | Y | 75 | **Good** |
| Velanovich (69) | 1999 | N | Y | N/A | Y | Y | Y | Y | Y | 75 | **Good** |
| Wilson (70) | 2005 | Y | Y | N/A | Y | N | N | Y | Y | 62.5 | **Poor** |
| Young-Afat (71) | 2019 | Y | Y | N/A | N | Y | Y | Y | Y | 75 | **Good** |
| Yusof(a) (72) | 2021 | Y | Y | N/A | Y | N | N | Y | Y | 62.5 | **Poor** |
| Yusof(b) (73) | 2021 | Y | Y | N/A | Y | Y | Y | Y | Y | 87.5 | **Good** |
| Zhao(74) | 2020 | Y | Y | N/A | U | Y | Y | Y | Y | 75 | **Good** |
| Y = Yes; N = No; U = Unclear; N/A = Not Applicable. | | | | | | | | | | | |

**Supplementary Figure 1**. Funnel plots for assessing risk of publication bias

**Supplementary** **Fig**. **1a** Funnel plot for analysis of effect of upper-body morbidity on quality of life (QOL) (Standardised Mean Difference (SMD)): Physical wellbeing


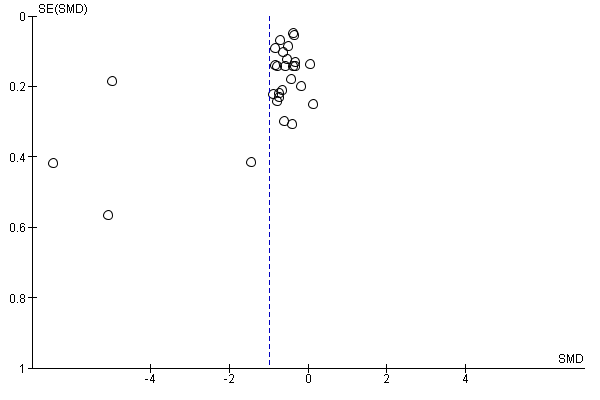


**Supplementary Fig. 1b** Funnel plot for analysis of effect of upper-body morbidity on QOL (SMD): Psychological/emotional wellbeing


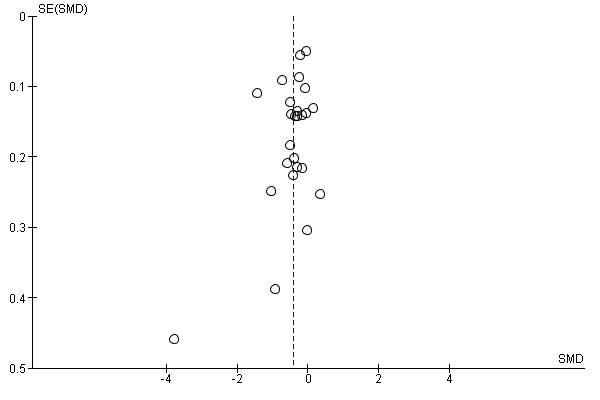


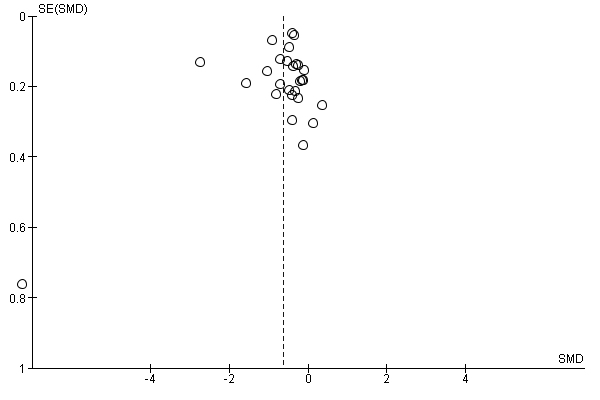


**Supplementary Fig. 1c** Funnel plot for analysis of effect of upper-body morbidity on QOL (SMD): Social wellbeing

**Supplementary Figure 2.** Sensitivity analyses


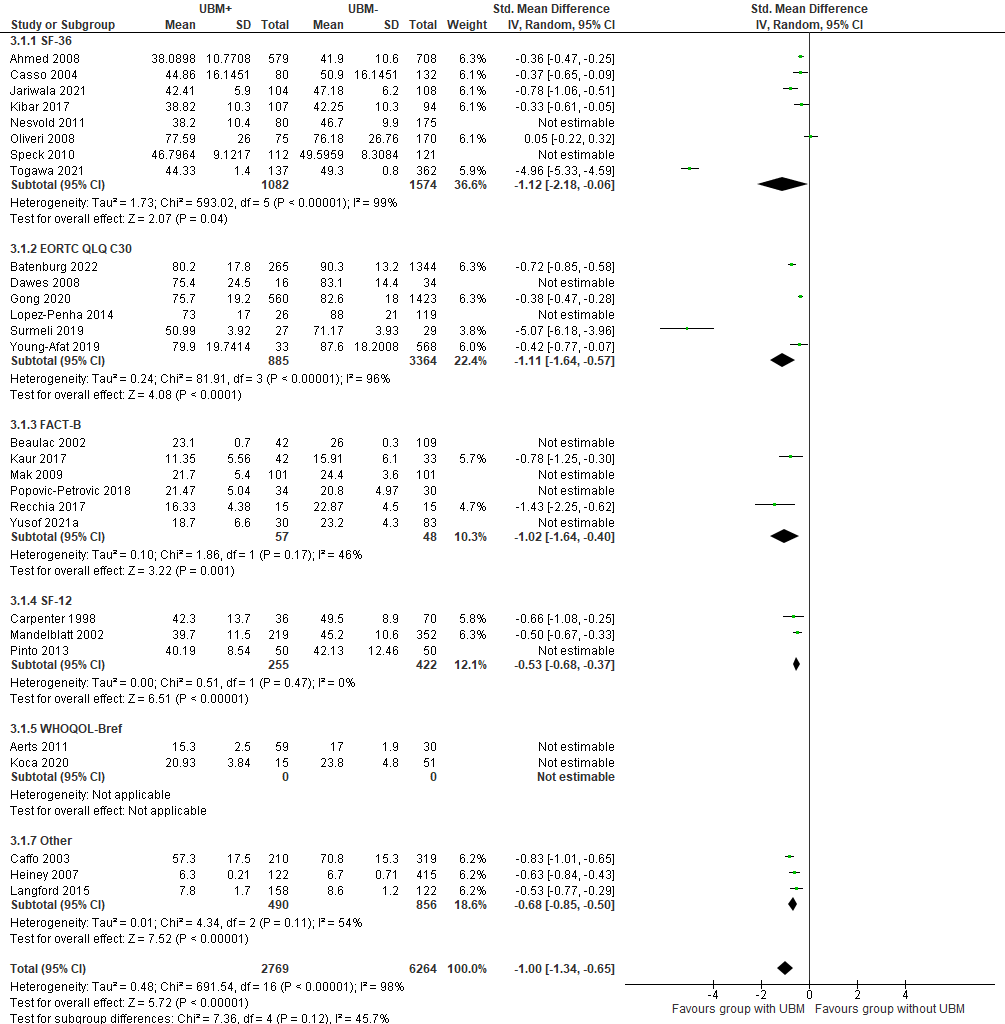


Effect of self-reported UBM on physical wellbeing (SMD)
IV, Random, 95%CI

**Supplementary** **Fig**. **2a** Sensitivity analysis: effect of self-reported upper-body morbidity on QOL (SMD): Physical wellbeing

**Supplementary** **Fig**. **2b** Sensitivity analysis: effect of self-reported upper-body morbidity on QOL (SMD): Psychological/ emotional wellbeing


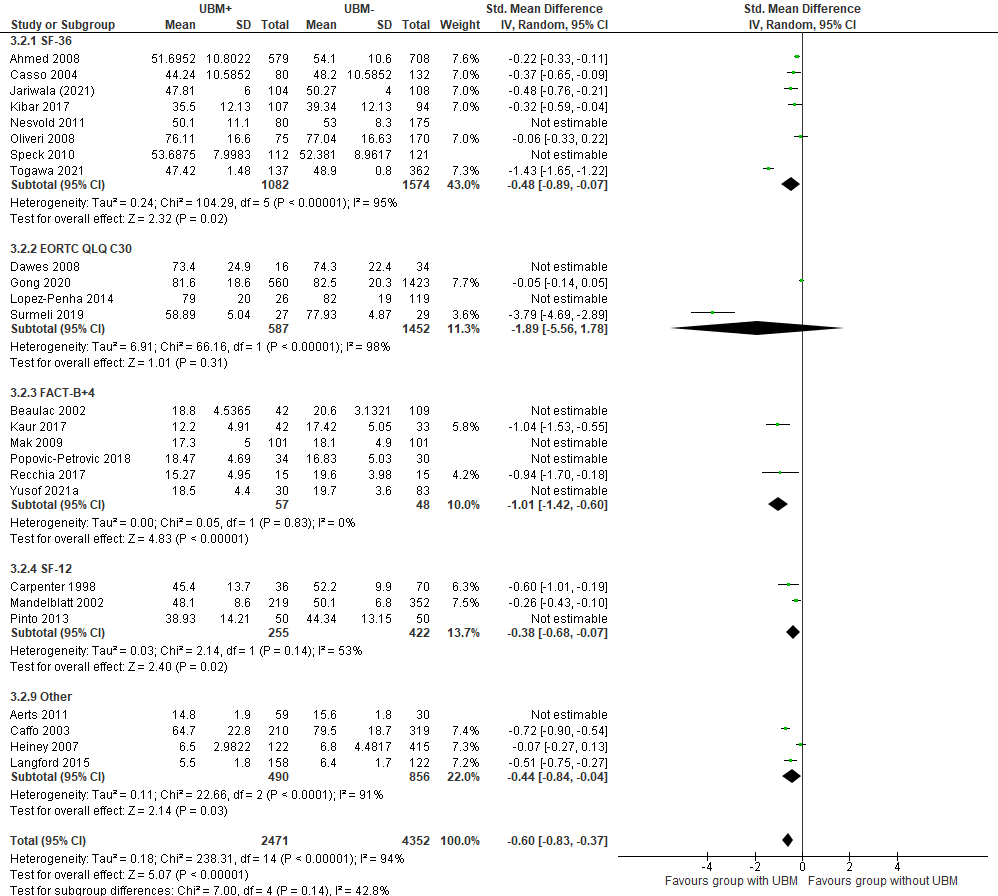


Effect of self-reported UBM on
psychological/ emotional wellbeing (SMD)
IV, Random, 95%CI


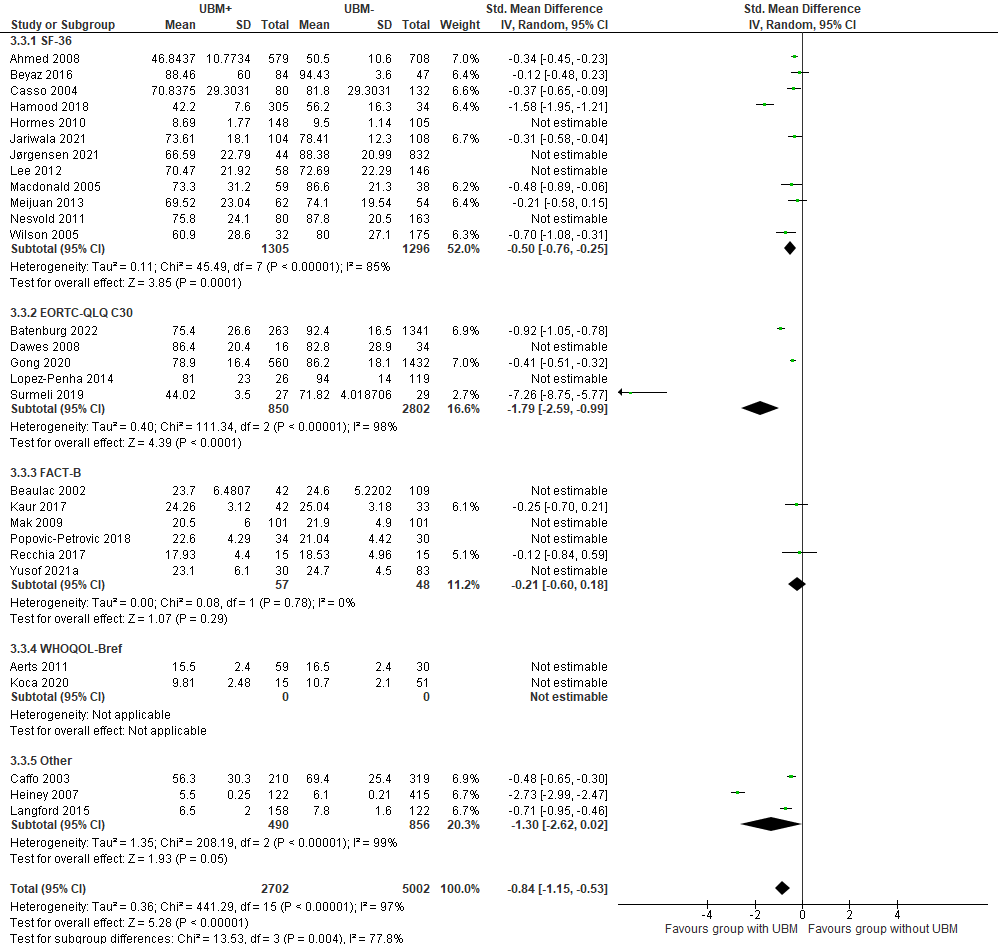


**Supplementary** **Fig**. **2c** Sensitivity analysis: effect of self-reported upper-body morbidity on QOL (SMD): Social wellbeing

Effect of self-reported UBM on social wellbeing (SMD)
IV, Random, 95%CI

**Supplementary Figures 3-7** Exploratory analyses: Effect of UBM on QOL questionnaire subscale scores (MD)

**3b**

**3a**


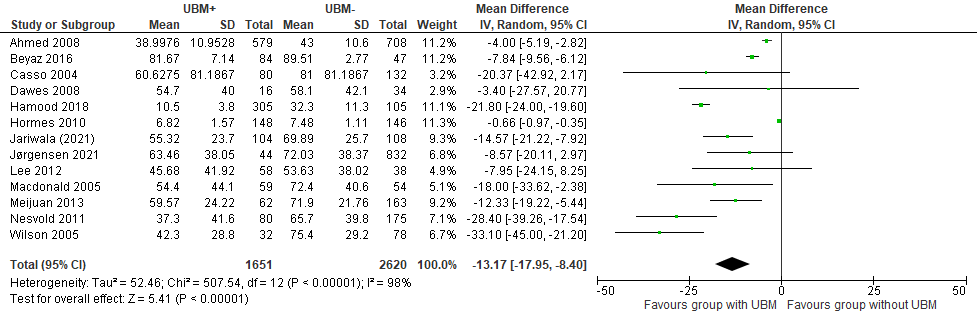

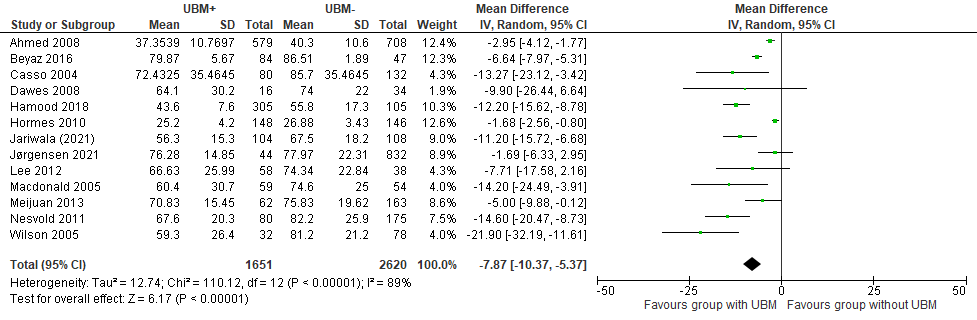


Effect of UBM on SF-36 physical function (MD)
IV, Random, 95%CI

**3c**


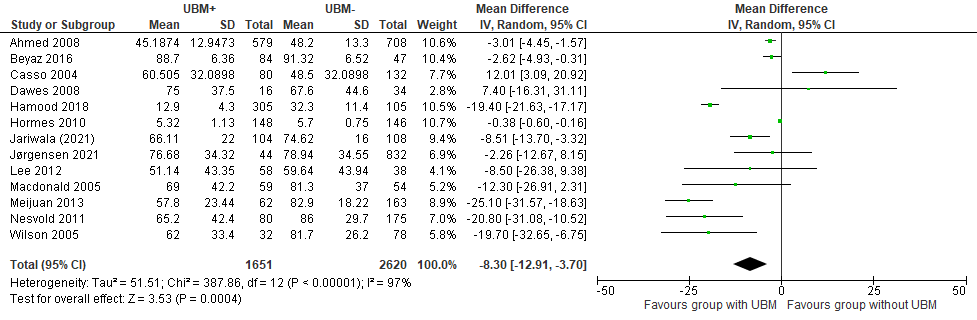


Effect of UBM on SF-36 role emotional (MD)
IV, Random, 95%CI


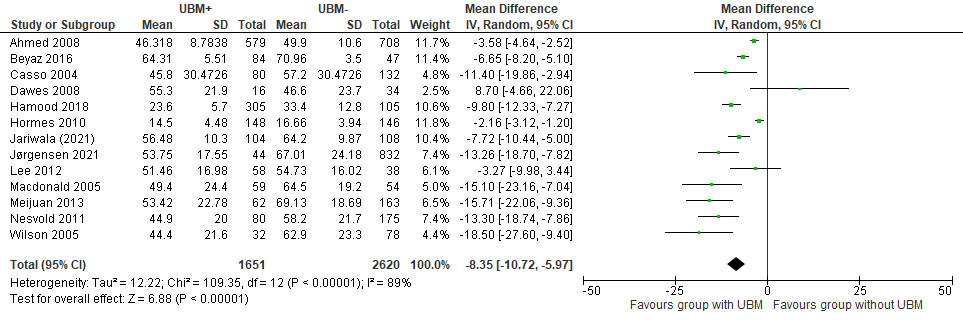


**3d**

Effect of UBM on SF-36 vitality (MD)
IV, Random, 95%CI

**3e**


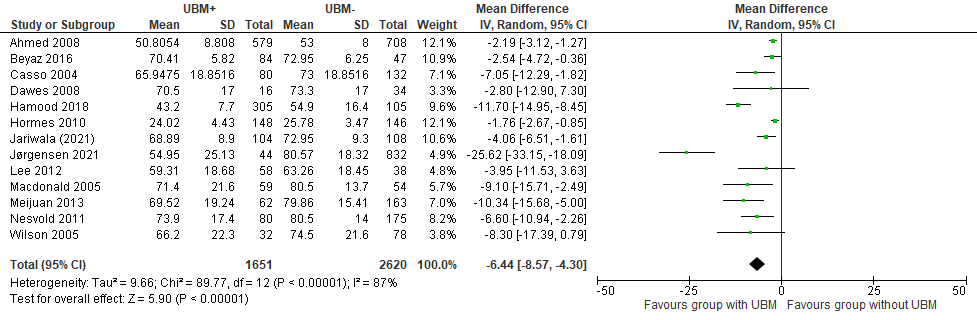


Effect of UBM on SF-36 mental health (MD)
IV, Random, 95%CI

**3f**


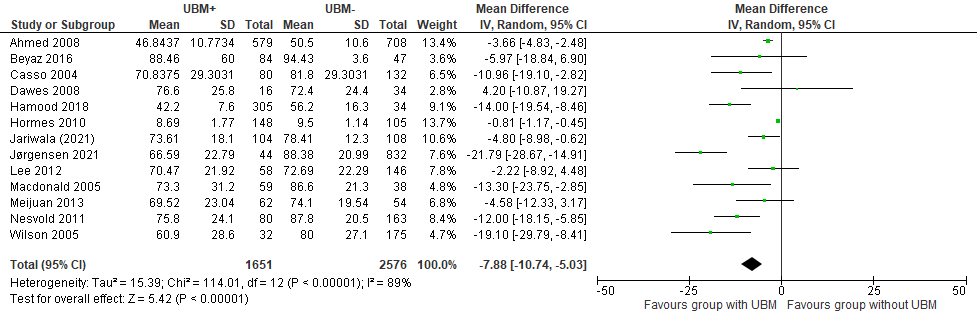


Effect of UBM on SF-36 social function (MD)
IV, Random, 95%CI


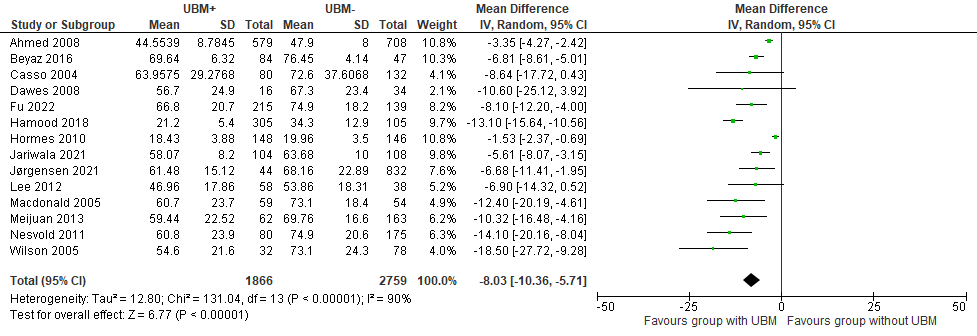


**3g**


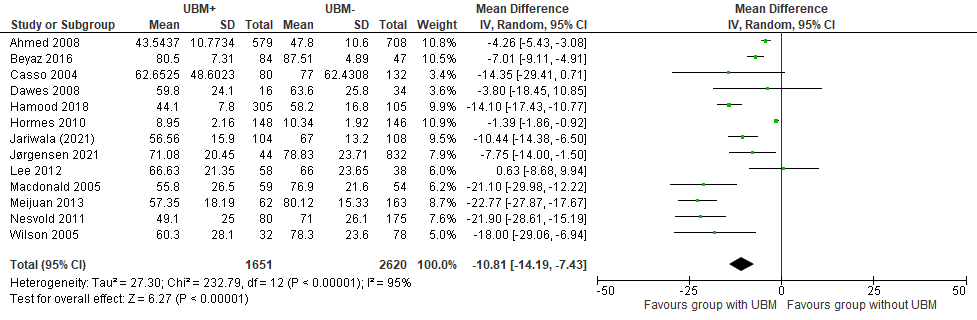


Effect of UBM on SF-36 bodily pain (MD)
IV, Random, 95%CI

**3h**

Effect of UBM on SF-36 general health (MD)
IV, Random, 95%CI

**Supplementary** **Fig. 3** Effect of UBM on SF-36 subscale scores: (3a) physical wellbeing, (3b) physical role functioning, (3c) emotional role functioning, (3d) vitality, (3e) mental health, (3f) social function, (3g) bodily pain, (3h) general health.


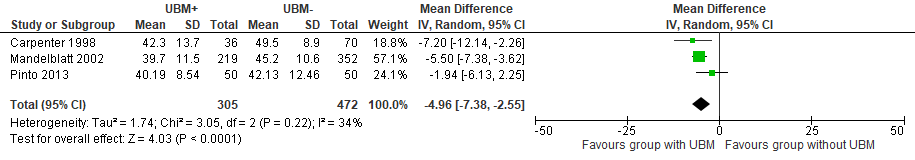


**4a**

Effect of UBM on SF-12 physical component score (MD)
IV, Random, 95%CI


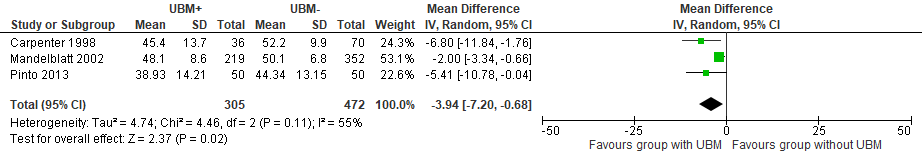


**4b**

Effect of UBM on SF-12 mental component score (MD)
IV, Random, 95%CI

**Supplementary** **Fig. 4** Effect of UBM on SF-12 subscale scores: (4a) physical component score, (4b) mental component score.


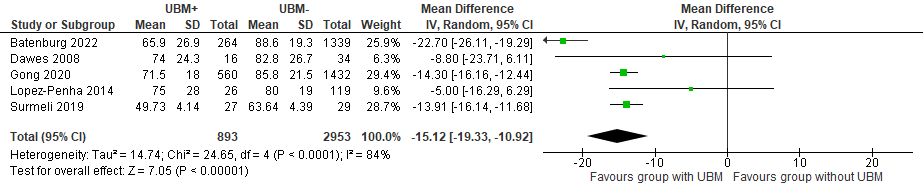

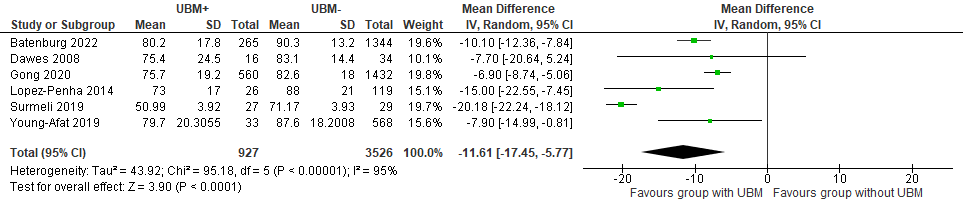

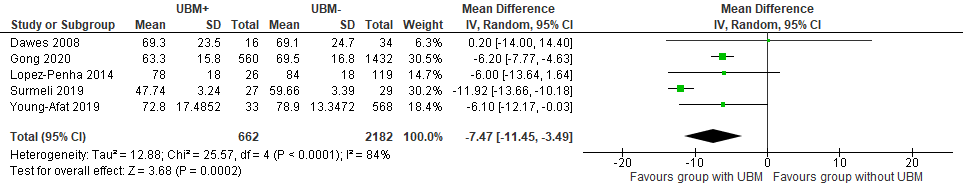


**5a**

Effect of UBM on EORTC QLQ-C30 global health status (MD)
IV, Random, 95%CI

**5b**

Effect of UBM on EORTC QLQ-C30 physical functioning (MD)
IV, Random, 95%CI

**5c**

Effect of UBM on EORTC QLQ-C30 role functioning (MD)
IV, Random, 95%CI


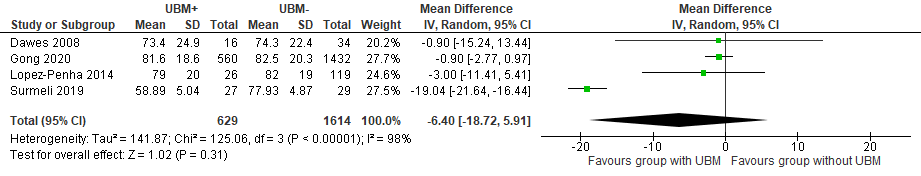


**5d**

Effect of UBM on EORTC QLQ-C30 emotional functioning (MD)
IV, Random, 95%CI


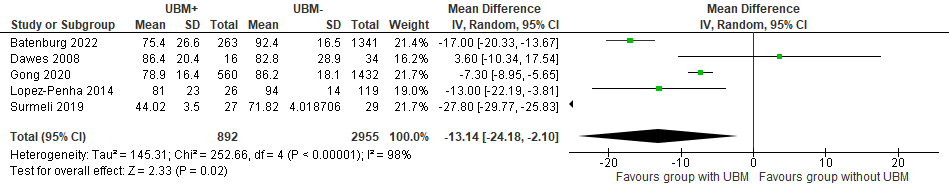


**5e**


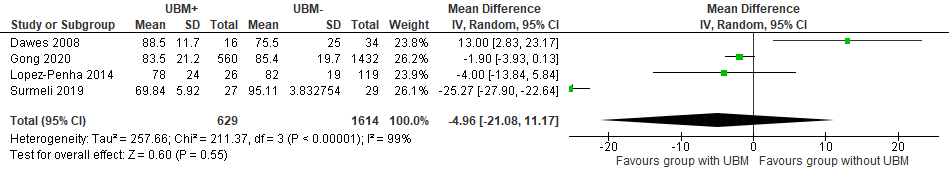


Effect of UBM on EORTC QLQ-C30 cognitive functioning (MD)
IV, Random, 95%CI

Effect of UBM on EORTC QLQ-C30 social functioning (MD)
IV, Random, 95%CI

**5f**

**Supplementary Fig. 5** Effect of UBM on EORTC QLQ-C30 subscale scores: (5a) global health status/ QOL, (5b) physical functioning, (5c) role functioning, (5d) emotional functioning, (5e) cognitive functioning, (5f) social functioning.

**6a**


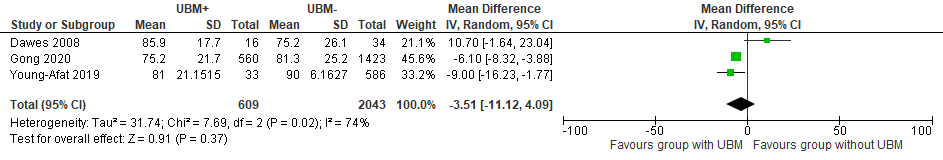


Effect of UBM on EORTC QLQ-BR23 body image (MD)
IV, Random, 95%CI


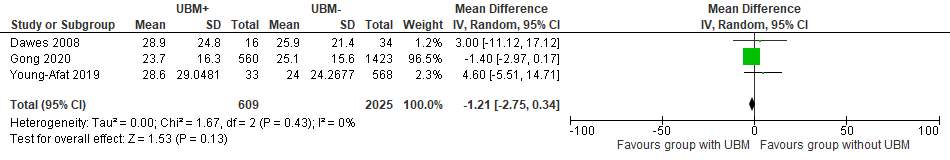


**6b**

Effect of UBM on EORTC QLQ-BR23 sexual function (MD)
IV, Random, 95%CI

**6c**


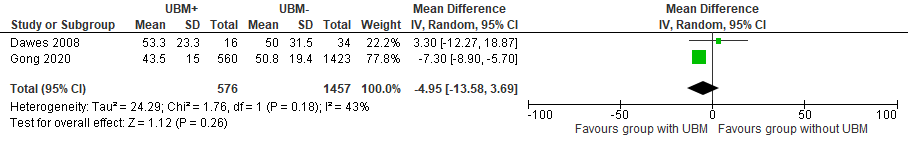


Effect of UBM on EORTC QLQ-BR23 sexual enjoyment (MD)
IV, Random, 95%CI

**6d**


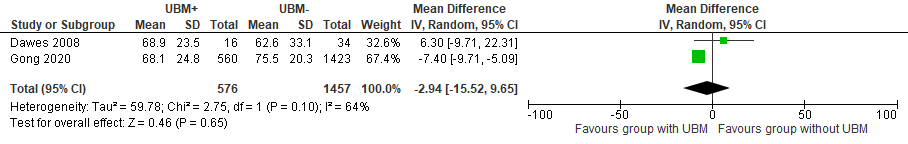


Effect of UBM on EORTC QLQ-BR23 future perspectives (MD)
IV, Random, 95%CI

**6e^**


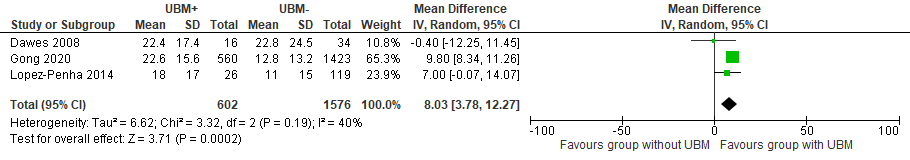


Effect of UBM on EORTC QLQ-BR23 breast symptoms (MD)
IV, Random, 95%CI

**6f^**


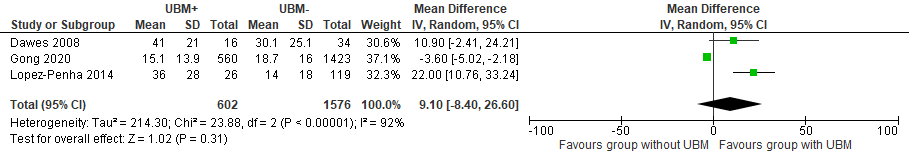


Effect of UBM on EORTC QLQ-BR23 arm symptoms (MD)
IV, Random, 95%CI

**Supplementary Fig. 6** Effect of UBM on EORTC QLQ-BR23 subscale scores: (6a) Body image, (6b) Sexual function, (6c) Sexual enjoyment, (6d) Future perspectives, (6e**^**) Breast symptoms, (6f**^**) Arm symptoms. **^** denotes subscale is reverse scored, with positive effect indicating higher/ worse symptom experience.

**7a**


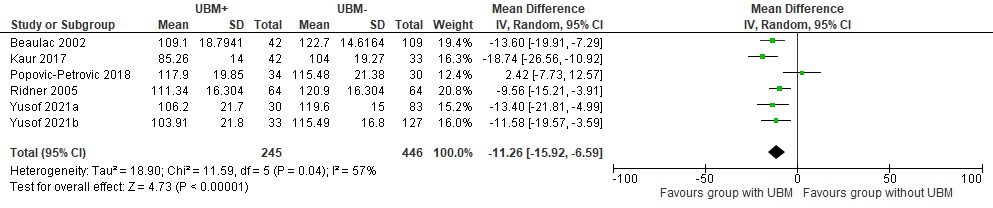


Effect of UBM on FACT-B total score (MD)
IV, Random, 95%CI

**7b**


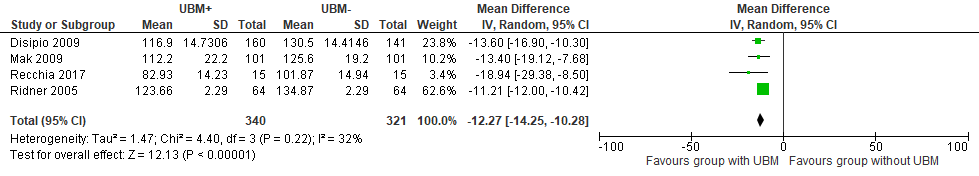


Effect of UBM on FACT-B+4 total score (MD)
IV, Random, 95%CI

**7c**


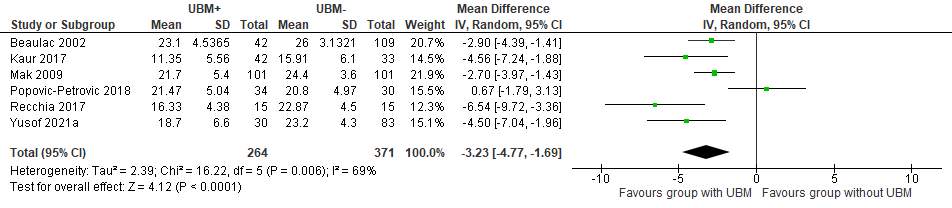


Effect of UBM on FACT-B physical wellbeing (MD)
IV, Random, 95%CI

**7d**


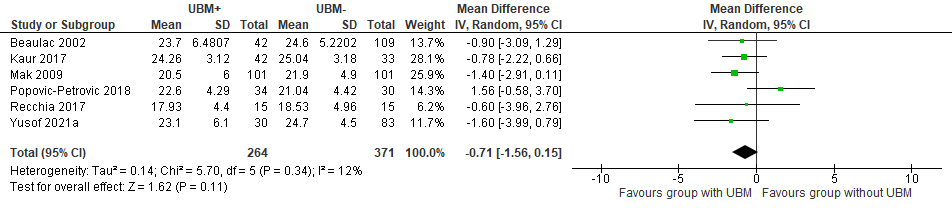


Effect of UBM on FACT-B social/ family wellbeing (MD)
IV, Random, 95%CI


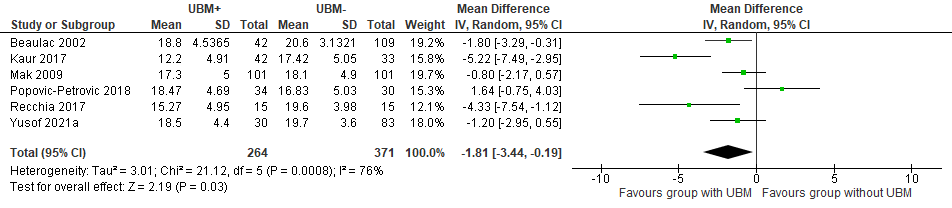


**7e**

Effect of UBM on FACT-B emotional wellbeing (MD)
IV, Random, 95%CI


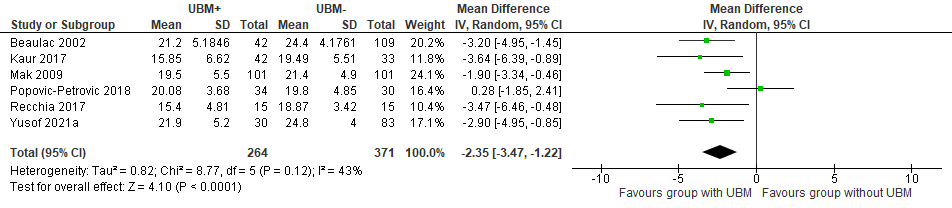


**7f**

Effect of UBM on FACT-B functional wellbeing (MD)
IV, Random, 95%CI


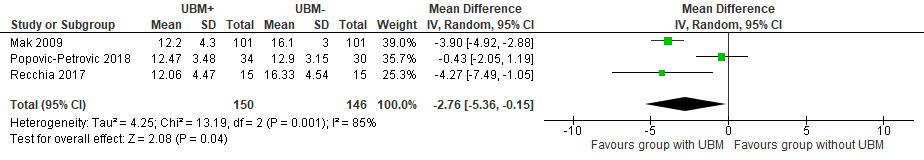


**7h**

Effect of UBM on FACT-B+4 arm symptom subscale (MD)
IV, Random, 95%CI

**Supplementary Fig. 7** Effect of UBM on FACT-B and FACT-B+4 total and subscale scores: (7a) FACT-B+4 total, (7b) FACT-B total, (7c) physical wellbeing, (7d) social/family wellbeing, (7e) emotional wellbeing, (7f) functional wellbeing, (7g) breast cancer subscale, (7h) arm symptom subscale.


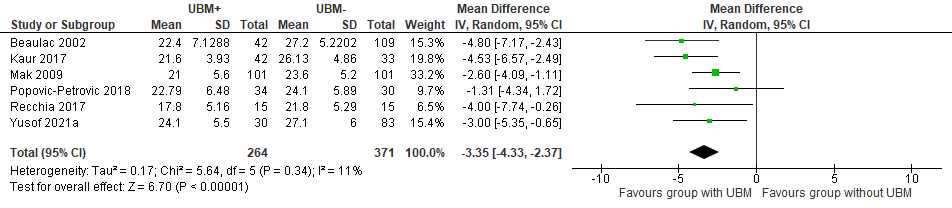


**7g**

Effect of UBM on FACT-B+4 breast cancer subscale (MD)
IV, Random, 95%CI


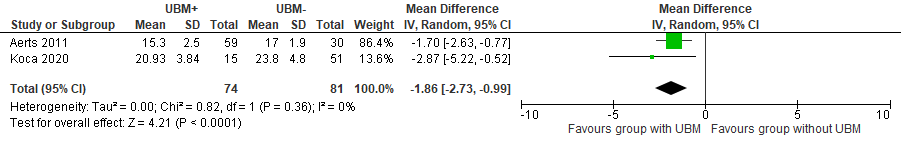


**8a**

Effect of UBM on WHOQOL-BREF physical health (MD)
IV, Random, 95%CI


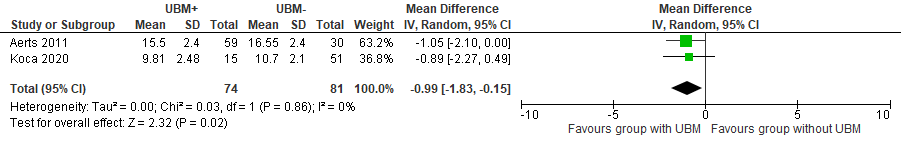


**8b**

Effect of UBM on WHOQOL-BREF social relationships (MD)
IV, Random, 95%CI


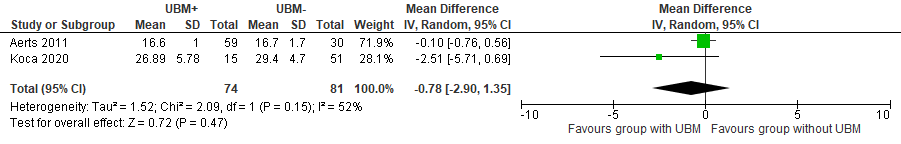


**8c**

Effect of UBM on WHOQOL-BREF environmental health (MD)
IV, Random, 95%CI


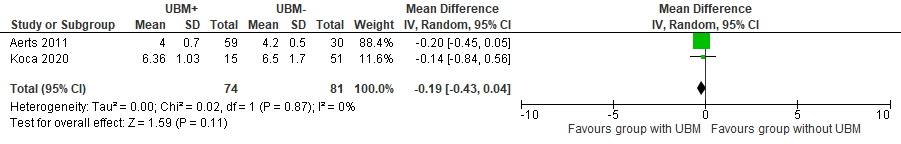


Effect of UBM on WHOQOL-BREF general health (MD)
IV, Random, 95%CI

**8d**

**Supplementary Fig. 8** Effect of UBM on WHOQOL-BREF subscale scores: (8a) physical health, (8b) social relationships, (8c) environmental health, (8d) general health.
